# Smart IoT-driven precision agriculture: Land mapping, crop prediction, and irrigation system

**DOI:** 10.1371/journal.pone.0319268

**Published:** 2025-03-18

**Authors:** Gourab Saha, Fariha Shahrin, Farhan Hasin Khan, Mashook Mohammad Meshkat, AKM Abdul Malek Azad

**Affiliations:** Electrical and Electronic Engineering Department, BRAC University , Dhaka, Bangladesh; Agricultural Sciences and Natural Resources University of Khuzestan, IRAN, ISLAMIC REPUBLIC OF

## Abstract

As the world population is increasing day by day, so is the need for more advanced automated precision agriculture to meet the increasing demands for food while decreasing labor work and saving water for crops. Recently, there have been many studies done in this field, but very few discuss implementing smart technologies to present a combined sustainable farming system. In this article, we present a complete integrated design of a smart IoT-based suitable agricultural land and crop selection, along with an irrigation system using agricultural mapping, machine learning, and fuzzy logic for precision agriculture. Multi-spectral band images from Landsat-8 satellite images of a chosen land are employed from USGS Earth Resources Observation and Science (EROS) Center for extracting indices that are used for agricultural analysis, determining the vegetation index, water index, and salinity index of that land using K-means. Furthermore, crop yield is predicted using Linear Regression and Random Forest, achieving accuracies of 93.49% and 95.87%, respectively, while using RMSE (Root Mean Squared Error) as the loss function. The LSTM model is used for healthy vegetation area forecasting highlighting the changes of the vegetation area over time. Such analysis helps to decide whether that land is suitable for farming or not. Multiple soil-parameter measuring sensors are used to identify suitable crop and fertilizer requirements for that land using IoT and machine learning. The ML model-based crop prediction showed 97.35% accuracy utilizing random forest algorithm. Finally, a fuzzy logic-based solar-powered irrigation system is used to monitor the water requirements of those crops and irrigate them according to their needs. The experimental results demonstrated that fuzzy logic has faster calibration rate of 66.23% and helps to save around 61% water in comparison to average logic algorithm. The implementation of a fuzzy logic algorithm significantly optimized water usage compared to traditional manual irrigation methods. These findings highlight the effectiveness of advanced computational techniques in enhancing agricultural practices and resource management.

## Introduction

Agriculture plays a pivotal role in food production and economic advancement, making significant contributions to social stability and public health. There are many developing countries like Bangladesh that are dependent on agricultural sectors [1]. According to the United Nations (UN), the global population is expected to reach 8.5 billion by 2030 and is forecast to increase to 9.7 billion by 2050 [2]. Consequently, the need for food production will progressively rise over time. However, due to continued degradation of the soil, loss of nutrients, climate change and inadequate infrastructure, the world might witness insufficient crop yield [1,3]. The majority of farmers in developing countries have a limited awareness of soil quality, including its existing nutrient levels and the necessary requirements to improve soil quality. Farmers, lacking prior information, are excessively applying fertilizers, insecticides, and irrigation in order to achieve higher crop yields [4]. This excessive usage depletes resources, degrades soil quality, and affects biodiversity.

Approximately 87.21% of the entire freshwater supply is allocated for use in the agricultural industry [5]. This highlights the unplanned utilization of water in irrigation. Energy is also an important factor in irrigation. Conventional water pumps used for irrigation are usually powered by either a diesel generator or grid energy. Both cases involve significant consumption of energy resources. This increases the risk of greenhouse gas (GHG) emissions, obstructing the goal of environmental sustainability. Therefore, sustainable agriculture is necessary to preserve soil quality, mitigate soil degradation, save water resources, and enhance biodiversity, all while preserving a natural and healthy environment. Implementing sustainable agriculture practices will result in the adoption of crop rotation, effective management of nutrient deficiencies in crops, control of fertilizer usage, and efficient water utilization for irrigation, ultimately contributing to a safer environment [6]. According to an economic survey revealed in 2018, there will be a drop of 25.7% of agricultural workers in the total workforce by 2050. Due to low per capita production, increasing costs in the agricultural sector and inadequate soil maintenance, people are migrating to occupations with higher benefits [6]. With the decline in the agricultural workforce, it is a suitable time to modernize the agriculture industry by implementing advanced technology.

Existing solutions often address isolated aspects of precision agriculture without offering an integrated approach. This study proposes a comprehensive IoT-driven precision agriculture system that integrates agricultural land mapping, crop selection, and a solar-powered irrigation system. Leveraging machine learning and fuzzy logic, the system offers a unified solution for sustainable farming practices. The novelty lies in its combined approach of multi-spectral satellite images, real-time soil monitoring, and intelligent water management, ensuring optimized resource utilization and enhanced crop productivity. This innovative approach bridges the gaps in current methodologies, providing a robust framework for addressing the challenges of modern agriculture.

## Literature review

A new approach using Landsat 8 data and random forest machine learning algorithm was developed by Pareeth et al. The approach introduced a hierarchical post-processing scheme to extract key land use land cover (LULC) types. HPF based data fusion technique is used to develop LULC maps at a spatial resolution of 15m. The final LULC map had an efficiency of 87.2% [7]. Kuo et al. proposed a system which is dependent on the variation of Deep lab V3 + . After testing the model could not show good accuracy due to the fixed value of the standard deviation gaussian filter [8]. Dhal et al. compared a real-time auto-regressive integrated moving average (ARIMA) model with a long short-term memory (LSTM) model. The results suggested the LSTM model achieved higher accuracy in comparison to ARIMA [9]. Arango et al. focused on the automatic delimitation of cultivable land with machine learning algorithms and satellite data. The Partitioning Around Medoids (PAM) algorithm was used here. PAM uses medoids as the centers of the clusters and these medoids are selected among the objects to be clustered. However, the research suggested developing feature selection techniques in order to improve the clustering [10]. Weiss et al. (2019) explore the applications of remote sensing in agriculture, emphasizing its role in monitoring crop growth, predicting yields, and supporting precision farming practices. The study suggested the use of satellites, UAVs and ground sensors in order to collect data. Advancement in sensor technology and cloud computing for data pre processing enhances the accuracy and applicability of remote sensors in agriculture [11].

For monitoring soil quality, it is required to collect different soil parameters. Multiple sensors are employed by Madhumati et al. for a real time soil monitoring system. A system was proposed which monitors the soil characteristics like moisture, nutrients, pH and soil temperature. In addition to that, it helped farmers in taking the right decision when applying fertilizer. This system helps in minimizing the usage of excess fertilizer and maximizing the yield [4]. A different approach is proposed by Palleveda et al. where a color sensor was used in order to obtain colors from the soil sample. Important information like soil pH, nutrient level was estimated using RGB values. The sensor collects the RGB value from the soil and compares it with the preexisting data. Afterwards, based on a deficiency of certain nutrients, the system suggests the required amount of fertilizer [12]. Ishak et al. focused on crop prediction by studying soil quality. Comparative analysis on three machine learning models: Naive Bayes, Logistic Regression and Random Forest was conducted in this study [13]. Another study integrated machine learning providing the best way of crop marketing from farmers to consumers [14]. After applying machine learning in crop prediction, Elsabi et al. suggested analyzing a wide range of data collected for farms, including IoT sensors to enable farmers to make more informed decisions about higher yield [15]. Saraswati et al. proposed smart agriculture and farming with live data such as temperature, soil moisture and humidity to monitor the surrounding environment. Micro controllers were used to collect data from different segments of the land. Afterwards, the data is transferred to Raspberry pi and sent to Things board server for data visualization [16]. In another research, environmental parameters are measured through multiple sensors. Later, the system decides and controls the suitable climate conditions for each crop. Message Queuing Telemetry Transport (MQTT) protocol is used for seamless data transmission to android applications [17].

Research on a novel intelligent irrigation system for deep learning neural network-based Internet of Things (IoT)-enabled intelligent irrigation system for precision agriculture (DLiSA) utilizes a deep-learning neural network and is enabled by the Internet of Things (IoT) [18]. This feedback-integrated system maintains optimal operation regardless of the weather conditions in any place and for any duration. The DLiSA model employs a LSTM network to forecast the volumetric soil moisture content for a one-day period, the irrigation period, and the spatial distribution of water needed to irrigate the arable land. The simulation results clearly demonstrate that DLiSA exhibits superior water management practices compared to state-of-the-art models within the experimental farming region. Another study on an irrigation system conducted by Bolu et al. utilized a master slave micro-controller configuration and pumps to accurately estimate the optimal timing for watering crops, considering the soil moisture level [19]. The system then delivers a regulated quantity of water to the crop roots. The technology as mentioned earlier is fully automated, operating continuously on solar energy, and is significant within the agricultural industry.

The conceptualization of an Internet of Things (IoT) enabled dynamic irrigation scheduling system (AgriSens) can aim at optimizing water utilization in irrigated agricultural regions. AgriSens offers instantaneous, automated, adaptable, and remote manual irrigation treatment for various stages of a crop’s growth cycle through the utilization of the Internet of Things. The experimental findings demonstrate that the AgriSens system enhances crop productivity by a maximum of 10.21% compared to the conventional manual irrigation approach. Additionally, it extends the lifespan of the network by 2.5 times compared to the current system, while maintaining a reliability of 94% even after 500 hours of operation [20]. Another research article outlines the functioning of the Internet of Things (IoT) node with the help of radiofrequency (RF). In addition, the RF energy harvesting approach on the IoT platform was utilized as an alternate method for supplying power to the platform’s IoT nodes. In order to achieve this objective, the construction and verification of a rectenna module designed for the purpose of RF energy harvesting was done and showed satisfactory performance [21]. A smart irrigation system utilizing the Global System for Mobile Communication (GSM) to assist farmers in effectively watering their agricultural areas was presented in this article [22]. This system transmits information including the soil’s humidity level, the surrounding environment’s temperature, and the motor’s state in terms of main power supply or solar power. The fuzzy logic controller is employed for the purpose of calculating input parameters such as soil moisture, temperature, and humidity, and generating outputs that indicate the state of the motor. Furthermore, the device automatically deactivates the motors to conserve electricity during periods of rainfall and uses solar panels to reduce power consumption drastically.

While each methodology individually contributes to various sectors of agriculture, there is a lack of a comprehensive solution. This article provides a smart IoT-driven precision agricultural system, which is a complete solution in the field of agriculture. The suggested approach addresses the issue of land selection and crop prediction fertilizer recommendation for increasing crop yield while conserving energy and water resources, thus providing a sustainable solution.

## Methodology

**Fig 1:**
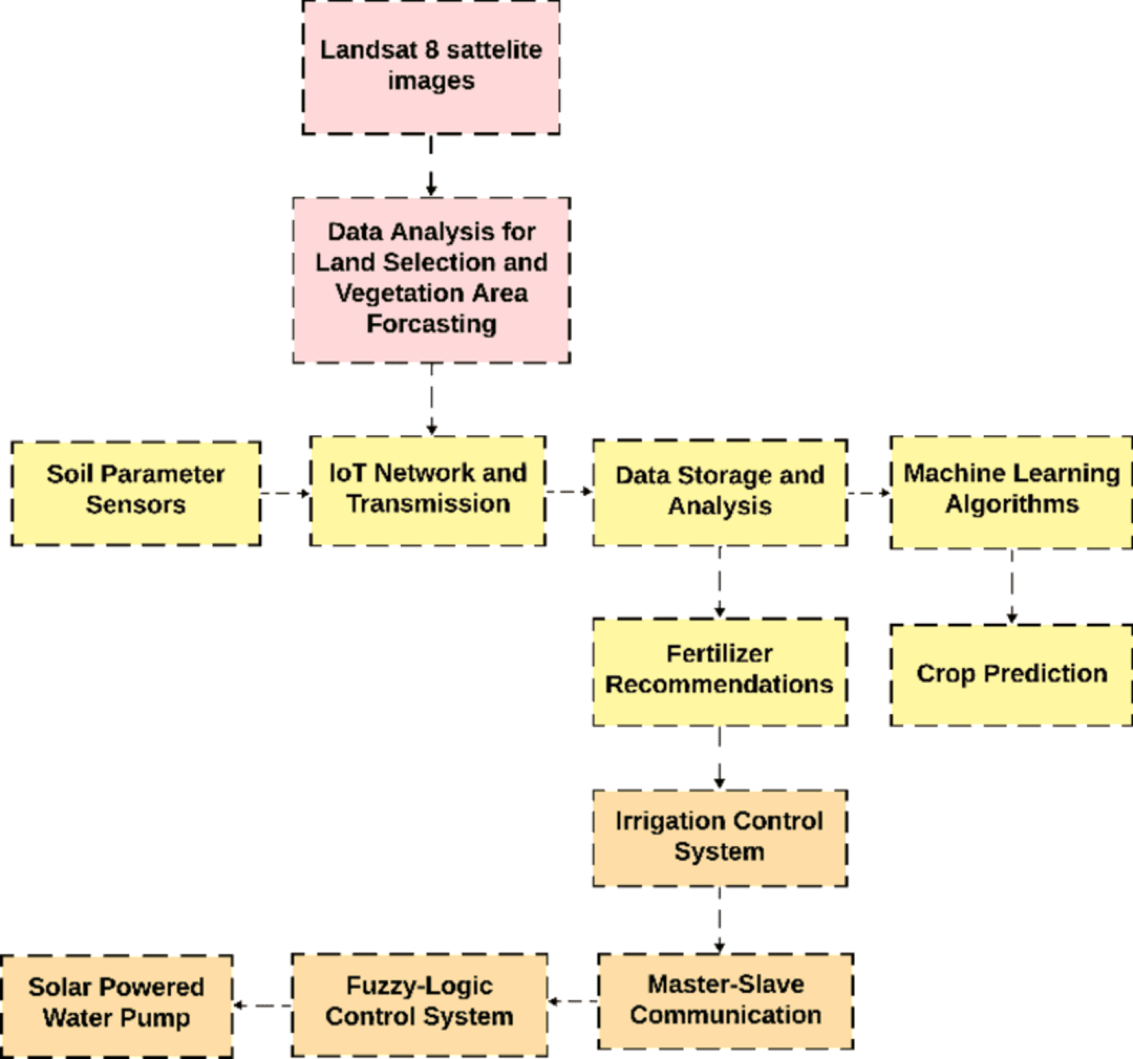
Block diagram of the proposed system.

This article introduces a precision agriculture system with an integrated design of a smart Internet of Things (IoT) infrastructure for the purpose of selecting appropriate agricultural land mapping, crop selection, fertilizer recommendation and efficient irrigation with the help of ML and Fuzzy Logic control systems. A complete block diagram of the proposed system is illustrated in Fig 1.

### C.1 Suitable land selection using agricultural mapping

The Landsat satellites provide earth observation data that can be utilized for vegetation analysis, soil moisture and temperature analysis, supporting precision agriculture. Our goal in this segment includes:

- Agricultural mapping and monitoring
- Remote sensing and image processing
- Machine learning for crop yield prediction
- Time series analysis for future predictions

**Fig 2:**
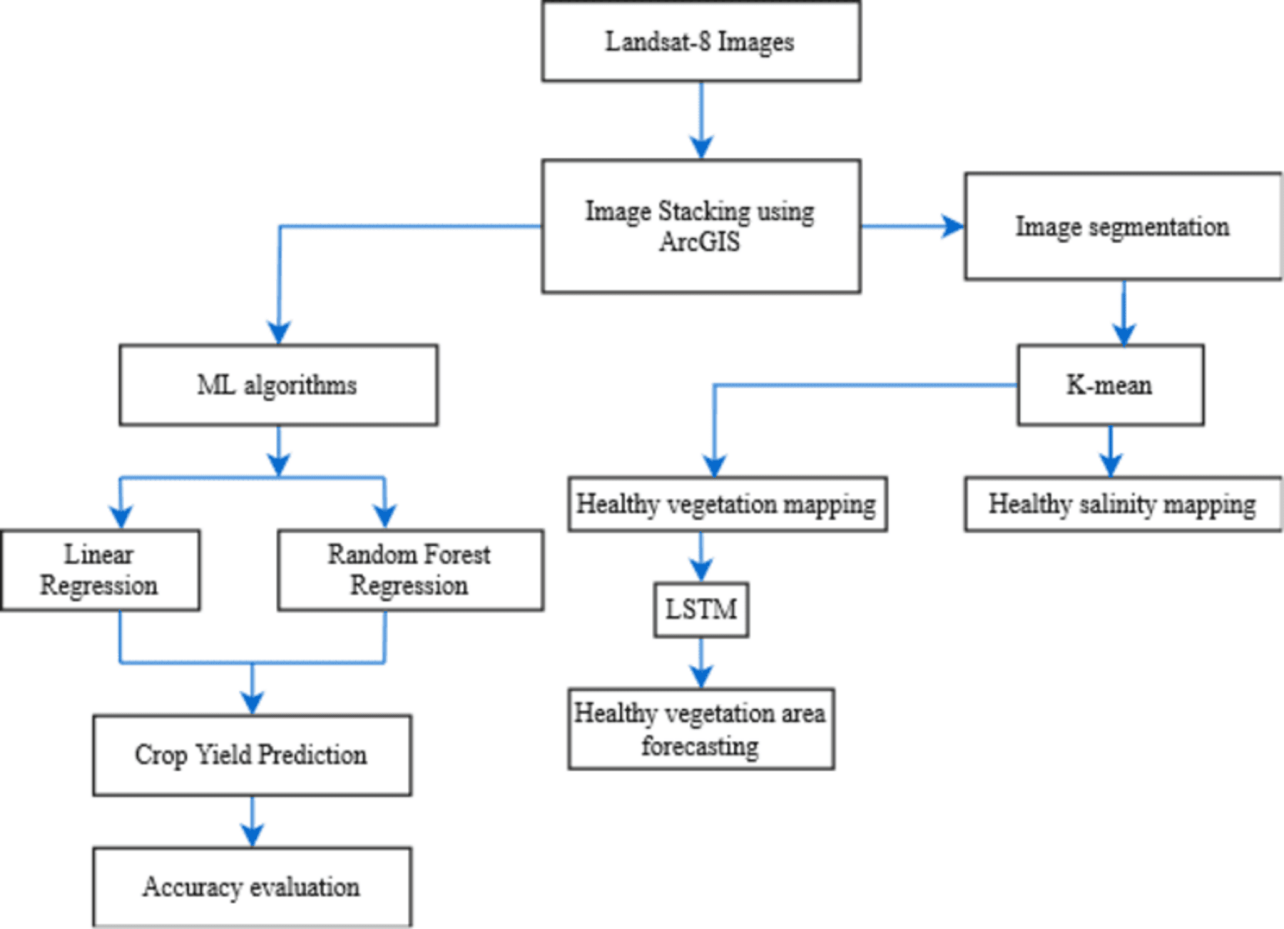
System diagram for agricultural land mapping using satellite image.


For the agricultural land study, multi-spectral band images from the Landsat-8 satellite were acquired using the USGS Earth Explorer, an online platform provided by the USGS Earth Resources Observation and Science (EROS) Center [23]. To further gather index information, ArcGIS is employed for image stacking. For directing focus on areas of healthy plants, the picture segmentation is done using K-means. Clusters must be identified, as this technique is capable of covering small areas and adapting to variations in the Normalized Difference Vegetation Index (NDVI), effectively capturing changes in vegetation health [24]. NDVI, analyzes the near infrared (NIR) and red spectral band. Furthermore, K-means was also utilized to identify high salinity areas with remote sensing Normalized Difference Snow Index (NDSI) to reveal which areas in that land need monitoring for higher yield, as high salinity contributes considerably to the loss of soil productivity. Based on this, the salinity of the land is categorized as high, moderate, and low from the image. For detecting salt effected area NDSI utilizes the unique spectral reflectance properties like the high reflectance in green band and low reflectance in short-wave infrared (SWIR) [25]. The formulas for NDVI and NDSI are presented in Eqs (1 and 2), respectively. The system diagram for agricultural land mapping using satellite image is presented in Fig 2.

$$\text{NDVI}=\frac{\text{NIR}-\text{Red}}{\text{NIR}+\text{Red}}$$ (1)

$$\text{NDSI}=\frac{\text{Green}-\text{SWIR}}{\text{Green}+\text{SWIR}}$$ (2)

To determine mapping of healthy vegetation, processed datasets of the land are used. Future healthy vegetation regions were predicted using the LSTM deep learning model to aid in future agricultural decision-making. Machine learning algorithms such as Linear Regression and Random Forest are used for crop yield prediction and accuracy of these models are evaluated using Mean Squared Error (MSE), Mean Absolute Error (MAE) and Root Mean Squared Error (RMSE) as shown in Eqs (3–5). These metrics are used for crop yield prediction because they measure how well the model’s predictions align with the actual values.


$$\text{MAE}=\frac{\sum_{i=1}^{n}\left|y_{i}-\hat{y_{i}}\right|}{n}$$ (3)

$$\text{MSE}=\frac{\sum_{i=1}^{n}{\left(y_{i}-\hat{y_{i}}\right)}^{2}}{n}$$ (4)

$$\text{RMSE}=\sqrt{\frac{\sum_{i=1}^{n}{\left(y_{i}-\hat{y_{i}}\right)}^{2}}{n}}$$ (5)

MAE provides straightforward average error magnitude, while MSE emphasizes large error due to squaring and used to detect significant inaccuracies. RMSE combines the benefits of MSE while retaining interpretability in the same unit as the targeted variable.

For research purposes, a dataset from 2014 to 2019 is used. Land parameters like soil moisture, chlorophyll, NDVI and NDSI are collected using the multi-spectral band from Landsat-8 satellite images [26,27].

### C.2 Crop prediction and fertilizer recommendation system

After finding and selecting land good enough for cultivation, next comes the need to choose suitable crops and fertilizer requirements on that land. A comprehensive analysis of seven key parameters, namely nitrogen, phosphorus, potassium, humidity in air, soil moisture, ambient temperature and soil pH, can facilitate the identification of the most suitable crop based on the existing soil nutrient levels. Our goals in this segment are as follows:

- Design a system for collecting soil parameters
- Develop an IoT-based system for real-time nutrient level monitoring
- Implement an algorithm to suggest suitable crops and fertilizer requirements for better yields

**Fig 3:**
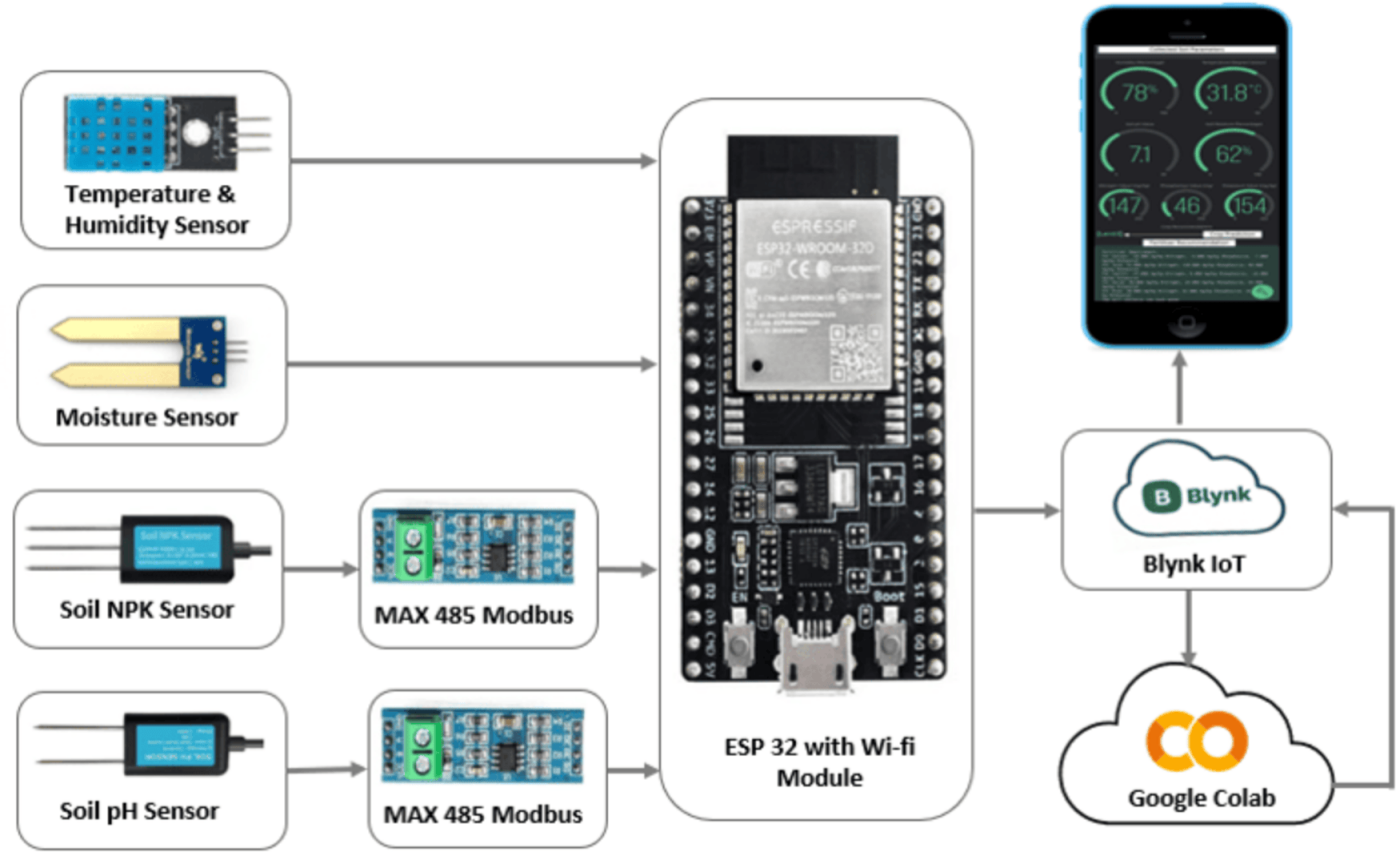
Block diagram for crop and fertilizer recommendations system.

In order to extract the soil parameters and data acquisition, using micro-controllers has been growing in popularity. These compact electronic devices serve as a crucial intermediary between the data source and the server, facilitating the seamless transmission of collected data. Once the data is successfully transmitted to the server, it is then processed and made available to the user in an easy-to-understand format, thereby enabling the user to access and interpret the collected values. Machine learning is used for predicting the most suitable crops for a particular land along with the fertilizer requirement [28]. The complete block diagram of the system is presented in Fig 3. Soil NPK sensors are utilized for measuring nitrogen, phosphorus, and potassium ions. A soil pH sensor that operates via probes is used to detect the pH levels in the soil. A moisture sensor is employed to measure the quantity of water present in soil. The DHT 11 sensor is utilized to measure the temperature and humidity in the environment of the selected land. Accuracy is critical for ensuring the reliability of agricultural monitoring systems. The soil NPK Sensor, NBL-S-NPK offers an accuracy of ± 2% full scale (F.S.), while the YL-69 resistive soil moisture sensor has an accuracy of up to ± 5%. For soil pH measurement, the NBL-S-PH sensor provides an accuracy of ± 1 pH, sufficient for analyzing soil chemical properties. Environmental parameters are monitored using the DHT11 sensor, which measures 20–80% relative humidity with ± 5% accuracy and 0–50°C temperature with ± 2°C accuracy. These sensors collectively ensure reliable data collection for crop and fertilizer recommendations while maintaining system feasibility and cost-effectiveness. These sensors collect the data from the soil sample, which goes to the processing unit of ESP 32 for data processing and storage in the cloud server. Blynk IoT serves as a monitoring system and for the purpose of data storage. Once the data is stored, it is consistently transmitted to Google Colab via an API. Subsequently, the gathered data is fed into the pre trained machine learning model [28].

A labeled dataset named ‘Crop Recommendation Dataset’ comprising features such as Nitrogen (N), Phosphorus (P), Potassium (K), soil pH, soil moisture, ambient temperature, and humidity was used for model training and testing [29]. Using a recognized dataset is crucial for ensuring the reliability of the research. The selected dataset, has been used in multiple reputable journal articles and research studies [30–32].

The research project incorporated multiple machine learning models to classify suitable crops based on soil and environmental parameters. The models included XGBoost, LightGBM, CatBoost, Decision Trees, Random Forest, Logistic Regression, Support Vector Machine (SVM), and K-Nearest Neighbors (KNN). Evaluation metrics such as accuracy, precision, recall, and F1-score were used to compare the models’ classification performance. The trained models were subsequently validated using both laboratory-acquired data and outdoor field data to ensure their applicability and reliability in real-world agricultural scenarios.

Once the soil characteristics have been inputted, a suitable crop is determined by comparing them with the pre-trained machine learning algorithm. Furthermore, the system employs the data collected by the sensor to accurately determine the optimal amount of fertilizer required to achieve maximum crop productivity. The fertilizer quantity is determined by comparing it to the threshold values in the ‘Fertilizer Recommendation Guide 2018’ [33]. As, the NPK sensor measures nutrient values in milligrams per kilogram(mg/kg), which may be a challenge when interpreting the quantity. Hence, the following equation can be employed to convert the unit to kilogram per acre (kg/ha) [34]. This conversion in Eq (6), facilitates a direct comparison with the values provided in standard fertilizer recommendation guides, ensuring accurate and practical fertilizer application. Here, bulk density is the mass of soil per unit volume. For our country perspective the unit the average bulk density of soil is considered from 1.0 to 1.4g/cm^3^. Again, the soil layer thickness for our case varies between 10 cm to 15 cm [28].


$$\frac{kg}{ha}=\frac{\frac{mg}{kg}\times Soillayerthickness\times Bulkdensity}{10}$$ (6)

### C.3 Solar-powered irrigation system

Even after suitable crop selection and optimal fertilizer implementation, irrigation has a significant importance for cultivation. Our goals in this segment are as follows:

- Increase agricultural production with an efficient algorithm for timely irrigation.
- Simplify irrigation by controlling every part of the system.
- Prevent mistimed irrigation to reduce water wastage and logging.
- Save electricity costs by using solar panels for renewable energy.
- Automate the controller to eliminate the need for manual operation and reduce mistakes.

The proposed system integrates renewable energy and utilizes automated and IoT technologies to facilitate a controlled and efficient water flow for irrigation purposes. It employs different algorithms to optimize water usage and achieve maximum efficiency.

**Fig 4:**
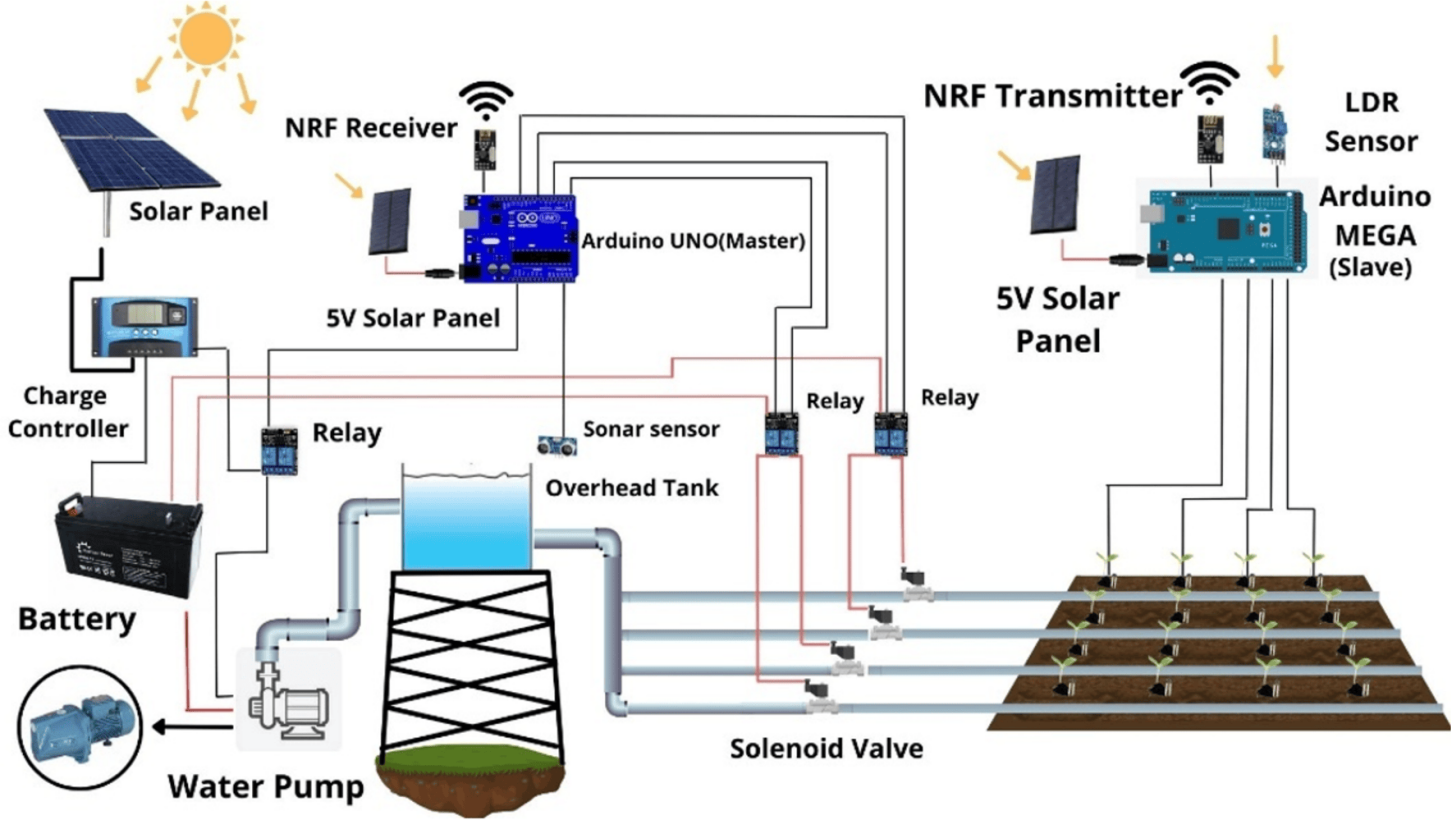
Block diagram for IoT-based irrigation system.

A smart irrigation system is presented in Fig 4. Here, solar energy is the driving source, and a battery is used as storage for ensuring continuous operation during low sunlight conditions. In the agricultural field, moisture sensors are deployed in selected areas based on the irrigation requirements. These sensors measure the soil moisture content and relay the data to the master micro-controller through slave micro-controllers. The slave micro-controllers act as intermediate nodes, collecting moisture data from the sensors and transmitting it to the master micro-controller for processing. If the soil moisture level falls below a preselected threshold, the master micro-controller activates the solenoid valve using a relay to start the irrigation process. The precise amount of water to be dispensed is determined using a Fuzzy Logic algorithm, which dynamically calculates the water requirement based on real-time sensor inputs. To evaluate the efficiency of the system, the results from the Fuzzy Logic algorithm are compared with an Average value-based algorithm. This comparison highlights the amount of water saved and the irrigation efficiency achieved by implementing advanced decision-making algorithms [35].

The proposed system ensures efficient water management by minimizing water wastage, optimizing energy use, and promoting sustainable irrigation practices, particularly in regions where water and power resources are limited.

#### C.3.1 Algorithm for the fuzzy logic controller.

**Fig 5:**
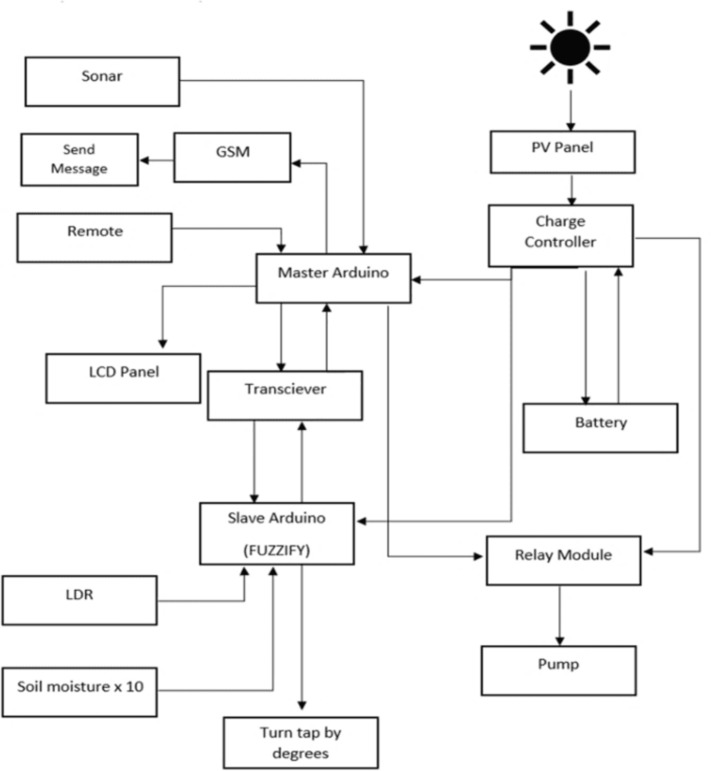
Block diagram for Fuzzy Logic-based irrigation system.

A flowchart demonstrating the monitoring of moisture in soil and analyzing water requirements for efficient irrigation of selected crops based on their specific requirements is presented in Fig 5. A stand-alone PV system along with battery storage is implemented for operating the water pump in a sustainable way. The design includes the master-slave configuration for communication in both the agricultural land and control system. Arduino MEGA is used as the master controller, whereas Arduino UNO is used as the slave controller. We introduce ten soil moisture sensors in order to run an Arduino-based system where the slave Arduino takes the values from the sensors and passes them on to the master Arduino with the help of a transceiver for decisions. It is flexible as the number of slaves can be increased by keeping only one master, i.e., the main Arduino. Stepper motors are used for running different lines of water as per the requirement with respect to the relay that receives water requirements information as input from the master Arduino. The size of the relay for this system depends on the size of the pump and the solar panel. The algorithm for this system is the inclusion of fuzzy logic in the master Arduino to dictate our solutions. The fuzzy logic system consists of fuzzy membership functions, and it gives us the output regarding the amount of valve to be opened based on the given moisture values. Here LDR (Light Dependent Resistor), is used so that the system is able to configure if it is day or night. The slave Arduino’s send signals as nodes to the main hub and the main valve is turned on. Therefore, the master Arduino is controlling the stepper motors to let water out as per requirement.

Due to financial viability and lower cost, nowadays an increasing number of people are getting interested in solar irrigation pumps with off-grid systems. Though our main work does revolve only around the controller, which is determining when irrigation is to required, our core component is also the pump that is serving to fill up a water tank whenever it needs to be fulfilled. The solar panel technology supports the farmers who are in the rural and isolated areas where only 25% people have access to electricity in Bangladesh. A study revealed that Bangladesh receives between 7 to 10 hours of sunlight daily, but this duration is reduced by 54% due to factors like rainfall, clouds, and fog, leaving only 4.6 hours of usable sunlight [36]. As a result, the abundant solar energy in Bangladesh presents a financially viable opportunity for various sectors by reducing reliance on traditional fossil fuels, which can be conserved for more critical applications instead of being used for tasks like water pumping. This shift not only supports environmental sustainability but also offers long-term cost savings. Therefore, the usage of a battery with a solar panel to back up the processing system and run DC loads such as the master and slave Arduino, and the relays, is designed here. The solar system makes sure to store the excess received power in a battery during the day so that it can also operate during the night from the battery when the solar panels cannot supply power. There can be usage of an MPPT system in order to make the direction and control of the current quite easier and make use of the maximum power output. This type of system is totally an off-grid system and therefore the whole system is self-sufficient and can be grown as per the requirements of the whole design. Overall, this is a closed-loop control system and therefore can be run completely unmanned.

**Fig 6:**
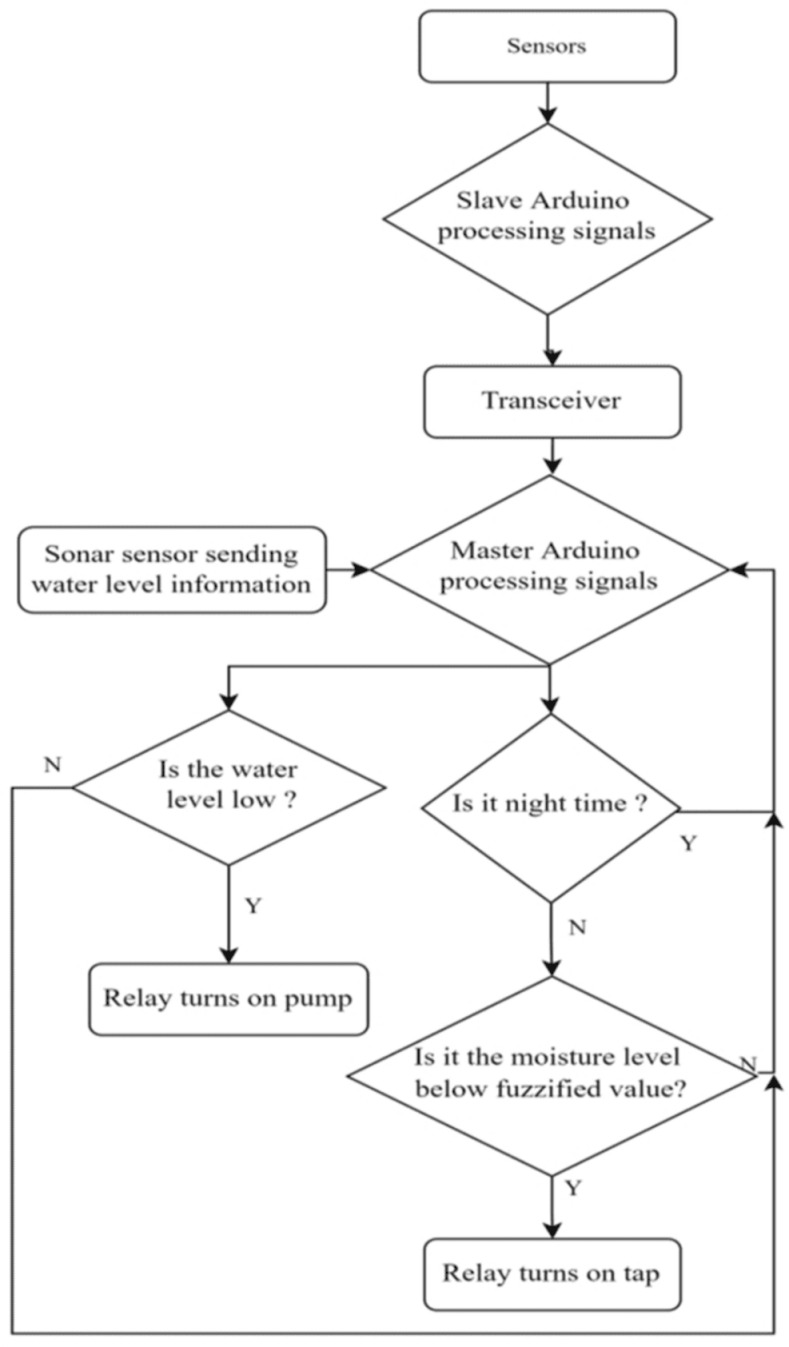
Flow chart of Fuzzy Logic based control system.

Fuzzy logic has the advantage of generating multiple values in between on and off states and has the ability to handle a huge variety of functioning conditions [37]. The fuzzy logic-based irrigation system algorithm has several crucial stages: initialization, fuzzification, inference, and defuzzification. First, language variables and phrases are established to represent input and output characteristics, including water level, time of day, soil moisture and pump status. Membership functions are subsequently created to depict the extent of membership for every input value inside these terms. A rule-based system is constructed as presented in Fig 6, by formulating if-then rules that define the correlation between input and output variables. During the process of fuzzification, the actual input data from the real world is transformed into fuzzy values. This transformation helps to determine the extent to which each input belongs to its respective fuzzy sets. During the inference stage, the fuzzy inputs are utilized to assess the rules, and the outcomes are combined to generate a solitary fuzzy output for each output variable. The defuzzification process ultimately transforms the aggregated fuzzy outputs into a precise, non-fuzzy numerical value, enabling practical decisions to be made such as, how much the solenoid valve to be opened. This method guarantees effective water utilization and ideal irrigation by intelligently adapting to prevailing conditions, considering water level, time of day, and soil moisture level [35].

#### C.3.2 Algorithm for average value controller.

**Fig 7:**
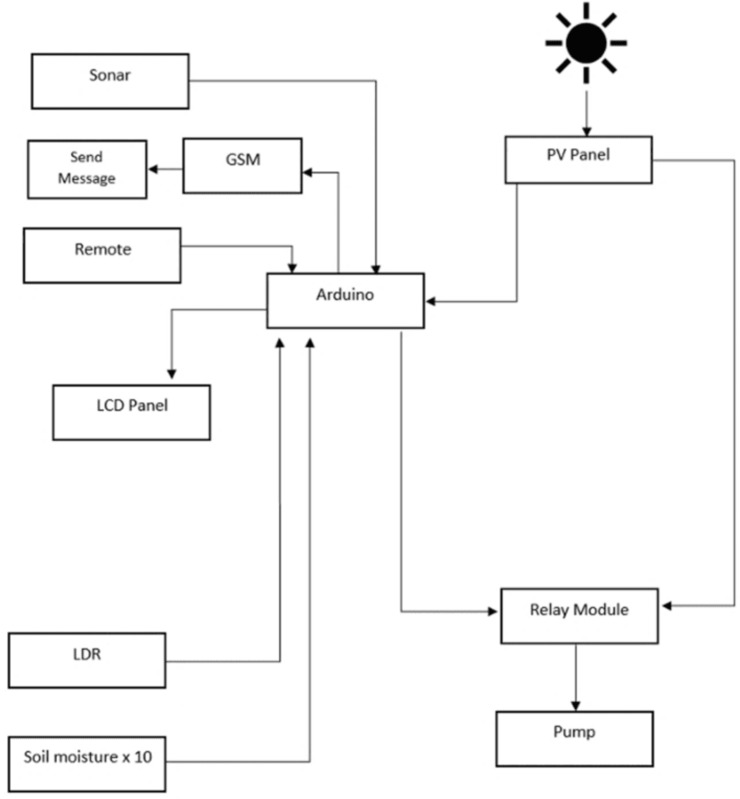
Block diagram for Average Value-based irrigation system.

An alternative design for our Fuzzy logic-based irrigation system is the Average value-based irrigation system as shown in Fig 7. The Arduino collects data from the sensors, calculates the average value, and regulates the release of water from the tank accordingly. So, instead of Fuzzy logic, the idea of average value is used here. This system is also a closed-loop control system and therefore can be run completely unmanned.

**Fig 8:**
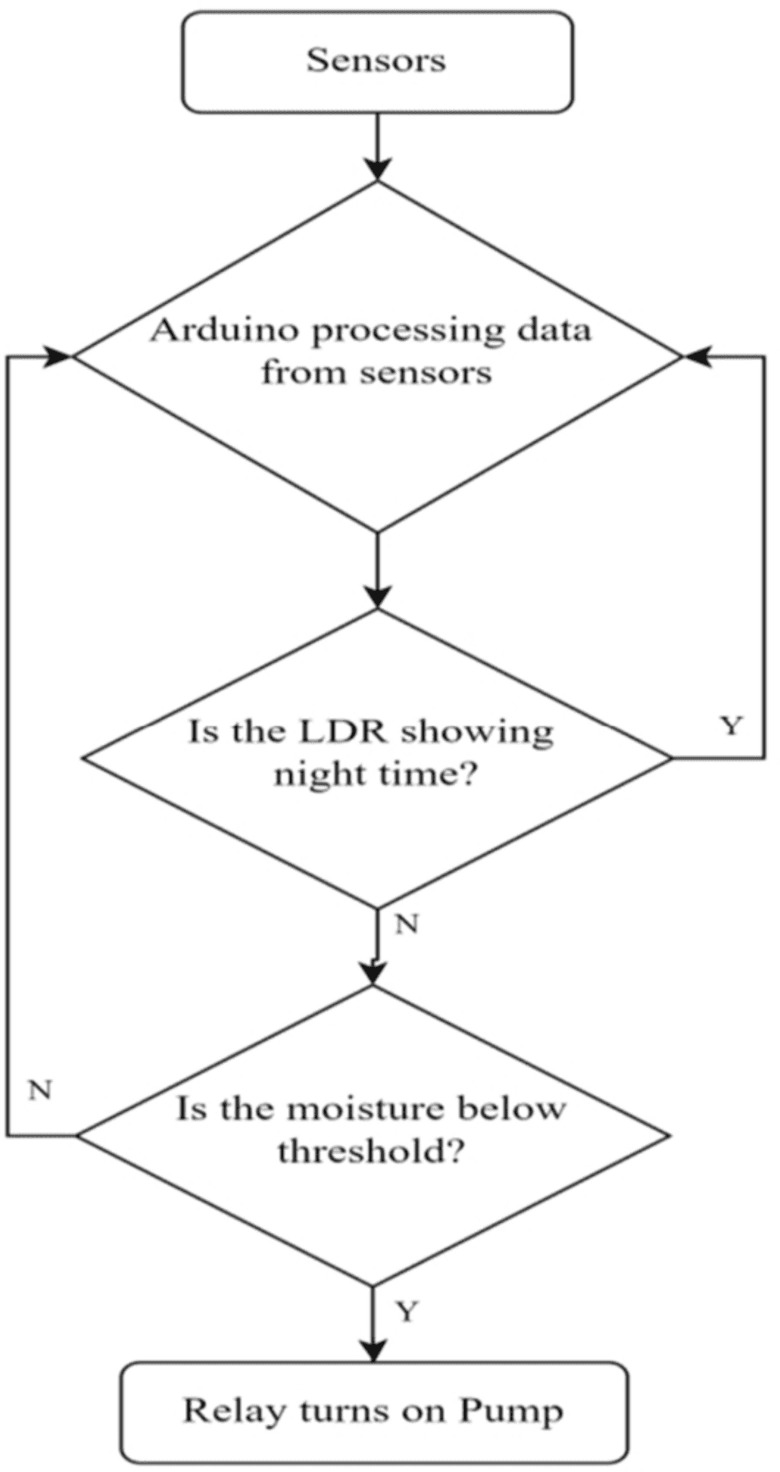
Flow chart of Average Value-based control system.

The complete flow chart of the Average logic algorithm is presented in Fig 8. The Average logic control system works on the principle of average value. Numerous sensors are connected with the micro-controller and the average values of all data are taken. Afterwards, the average value is compared with the preset threshold value. While the moisture value is below threshold, the stepper motor opens the solenoid valve for water flow.

#### C.3.3 Water requirement calculation for irrigation system.

The system considers water management and should make the lowest reduction in water wastage. Water is a valuable resource and therefore making sure that the water is not wasted for unnecessary reasons is a must. The trustworthy factor in the case of finding out the amount of water required for any size of land is the ET_o_ factor, which is calculated based on some parameters. The basic formula for the calculation of crop water requirements is:


$$ET_{crop}=kc\times ET_{o}$$ (7)

Here, ET_crop_ is the water requirement of a given crop in mm per unit of time, kc indicates crop factor and ET_o_ means the reference crop evapotranspiration in mm per day [38]. We aim to ultimately find the ET_crop_ value to determine water requirement. Table 1 shows the crop factors for different crops.

**Table 1:** Crop factor of different crops according to height.

Crop	Kc (initial)	Kc (medium)	Kc (end)	Max Height (m)
Corn	0.8	1.15	0.15	1.5
Sugar Cane	0.4	1.25	0.75	3
Rice	1.05	1.2	0.9	1
Tomato	0.45	1.15	0.8	2
Cucumber	0.45	0.9	0.75	1.5

To find ET_o_, the formula to be used is [39]:


$$ET_{o}=\frac{0.408\Delta \left(R_{n}-G\right)+\gamma \frac{900}{T+273}u_{2}\left(e_{s}-e_{a}\right)}{\Delta +\gamma \left(1+0.34u_{2}\right)}$$ (8)

where Rn = net radiation at the crop surface [MJ m day-]
G =  soil heat flux density [MJ m’ day ‘]
T = air temperature at 2 m height [°C]
u_2_ = wind speed at 2 m height [m s-1]
es = saturation vapor pressure [kPa]
e_a_ =  actual vapor pressure [kPa]
e_s_ - e_a_ = saturation vapor pressure deficit [kPa]
*Δ* = slope vapor pressure curve [kPa °C-1]
*γ* = psychrometric constant [kPa °C-1]

ET_o_ varies from place to place. According to research from Bangladesh Agricultural University, the ET_o_ value of Mymensingh District of April is 5.5. Since this irrigation system was being analyzed in Mymensingh, we considered the water required as per the ET_o_ of Mymensingh. kc of tomato in middle growth is =  1.15 (average among different kinds of them). So, ET_crop_ (for tomato) is 6.32 (5.5 * 1.15). Therefore, a 100m^2^ area of land with tomato requires: 10 ×  10 ×  0.0063 =  0.63 m^3^ =  630 liters of water per day for Fuzzy logic. This was done for a draft field and so we are to calculate the size of our field and based on that field we are to calculate the amount of water saved for our irrigation system by comparing it with the Fuzzy logic-based system. A study conducted on a 4m² test bed for rice cultivation found that 26.4 liters of water were required daily. Theoretically, for a 100m² field, the daily water requirement was calculated as 10 x 10 x 0.0066 =  0.66m3 =  660 liter/per day. During irrigation tests conducted over three separate days using a device with a multi-step water flow output, the water needed was measured. The results showed that the device reduced water usage by approximately 12.3% while maintaining the required moisture level. This saved about 81 liters of water compared to the estimated daily requirement for a 100m² field [40].

The research work required no permits as the initial testing were conducted in the laboratory of our institute. Since the research focusses on soil data collection, this does not raise any ethical or regulatory concerns. As the device is portable, no additional setup is required and the field testing was carried out on public land and for the irrigation testing, a small-scale model is prepared in and the impact was calculated in broader scale.

## System implementation

### D.1 Prototype for crop and fertilizer recommendations system

**Fig 9:**
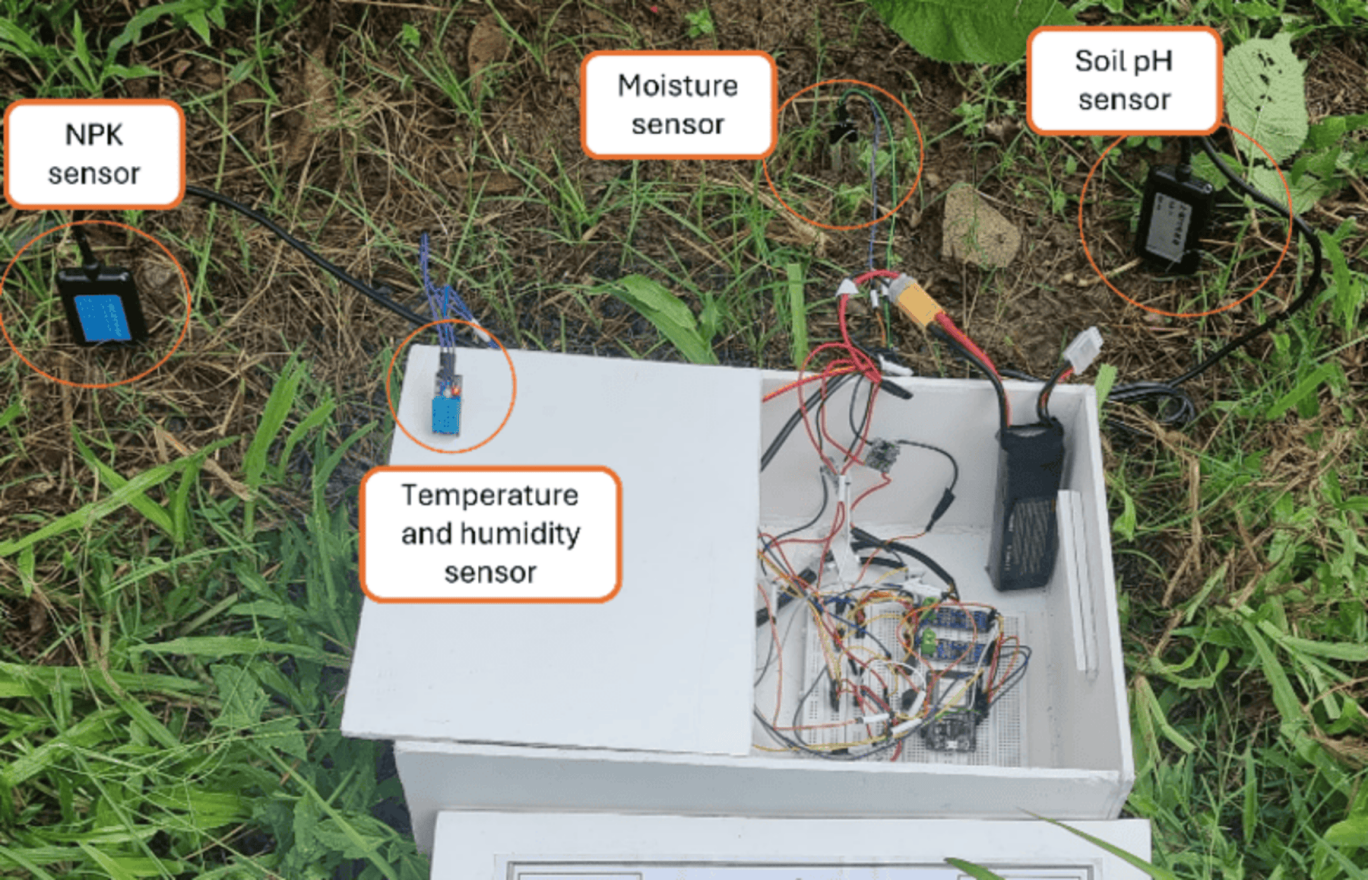
Prototype model for crop and fertilizer requirements.

The prototype of the soil parameters collecting system is displayed in Fig 9. The right side of the device is equipped with all the sensors, while the left side is dedicated to housing the circuits. To simplify the prototype testing process, five crops were selected from the dataset for field testing, as not all crops are suitable for cultivation in our region. The chosen crops include rice, jute, cotton, maize, and lentils. Table 2 displays the average values of the parameters for specific crops as per provided in the crop recommendation dataset [29].

**Table 2:** Mean parameter values for the selected crops.

Parameters	Cotton	Jute	Lentil	Maize	Rice
**Temperature (°C)**	31.23	26.11	35.87	32.83	32.61
**Humidity (%)**	58.90	82.49	58.08	77.03	70.25
**Soil pH**	6.3	7.0	7.1	5.9	5.6
**Nitrogen (mg/kg)**	102.35	203.04	130	188.66	163.77
**Phosphorus (mg/kg)**	51.70	189.69	37.70	66.27	112.77
**Potassium (mg/kg)**	92.55	201.49	139.32	198.62	134.26

To verify the reliability of the system used to determine the suitable crops and fertilizer requirements for a certain land region, three soil samples were obtained from Tangail, Barguna, and Khulna within Bangladesh. Subsequently, the samples were examined to evaluate the soil conditions and determine the optimal crops and fertilizers for each specific area. Later, an external location was selected to carry out real-life experiments.

### D.2 Prototype design for irrigation system

**Fig 10:**
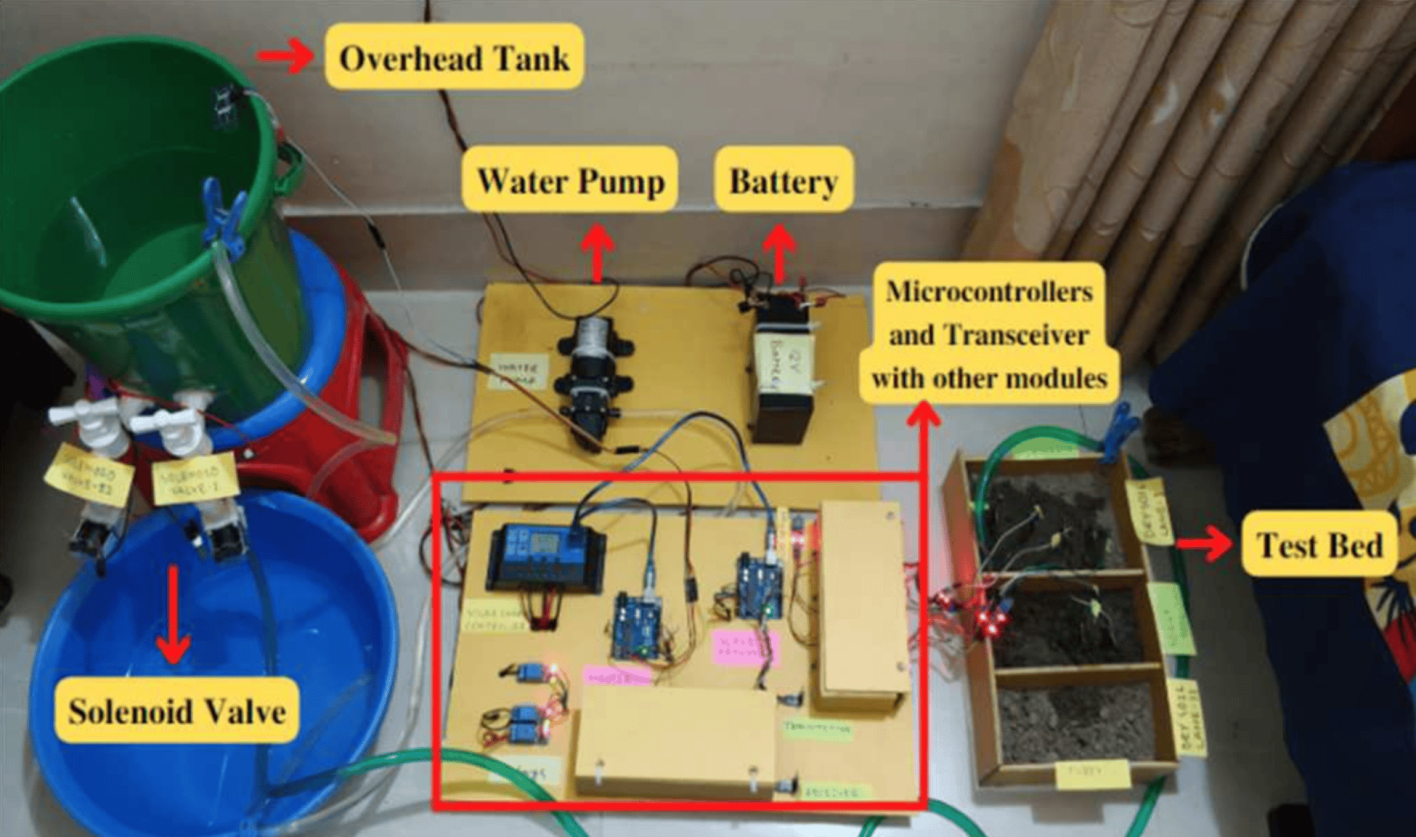
Hardware design for IoT-based irrigation system.

The comprehensive design of the hardware configuration for the solar-powered irrigation system is presented in Fig 10. It incorporates both Fuzzy logic and Average value methods to autonomously monitor and determine the water requirements for crops, and subsequently irrigate them. Instead of solenoid valves, the tap is connected to the water storage. To ensure perfect readings, hundreds of sensors may be needed which may not be feasible here. So, as shown in the diagram, for each section of land, there will be one or two sensors planted on one side of the land and on the opposite side water pipe will be planted with a motor-controlled water tap. That solenoid valve can be operated in either single-step output or multi-step output.

In a single step, the valve or tap will be shut off instantaneously using the Average based algorithm; in multi-step, the valve will be shut off step by step utilizing the Fuzzy logic algorithm. For example, when the moisture level reaches near to the required level, the tap position will be close to the off state and when the moisture level finally reaches the required level, the tap will be completely shut off, letting no more water out.

The primary objective of this system is to reduce consumption of water and electricity, enhancing agricultural output and reducing the labor required by farmers. The incorporation of these properties makes this technique a viable choice for improving agricultural and irrigation efficiency. The water pump utilizes solar energy to transfer water into the tank, where it is stored until the above water tank reaches its maximum capacity. The sonar sensor serves as an input to the control unit, allowing it to determine if the tank is at maximum capacity. Based on this information, the controller controls the operation of the water pump through a relay. The master controller adjusts the status of the valve based on the moisture level of the land. The duration for which the solenoid will be activated can be determined using either fuzzy logic or the average value algorithm. The solar panels provide energy to operate the water pump, micro-controllers, and relays.

To validate the system, we performed trials on a bed that was fitted with six sensors and two rows of solenoid valves. The samples of both saturated and dried soil were gathered and deposited into a separate soil bed. Subsequently, moisture sensors were inserted into each soil sample separately in order to evaluate the outcomes. The tap would be activated, enabling the water to stream from the bucket into the soil bed. When the moisture level of soil is close to the threshold, the water flow from the tap is discontinued. The fuzzy system ensured that each tap supplies the required amount of water. The entire system operated solely on solar energy. To apply this on a broad scale in a real-world scenario, it would be necessary to use a bigger water pump paired with a water tank, along with extra sensors and valves, to handle multiple soil segments.

## Result and performance analysis

### E.1 Suitable land selection

#### E.1.1 Healthy vegetation mapping.

**Fig 11:**
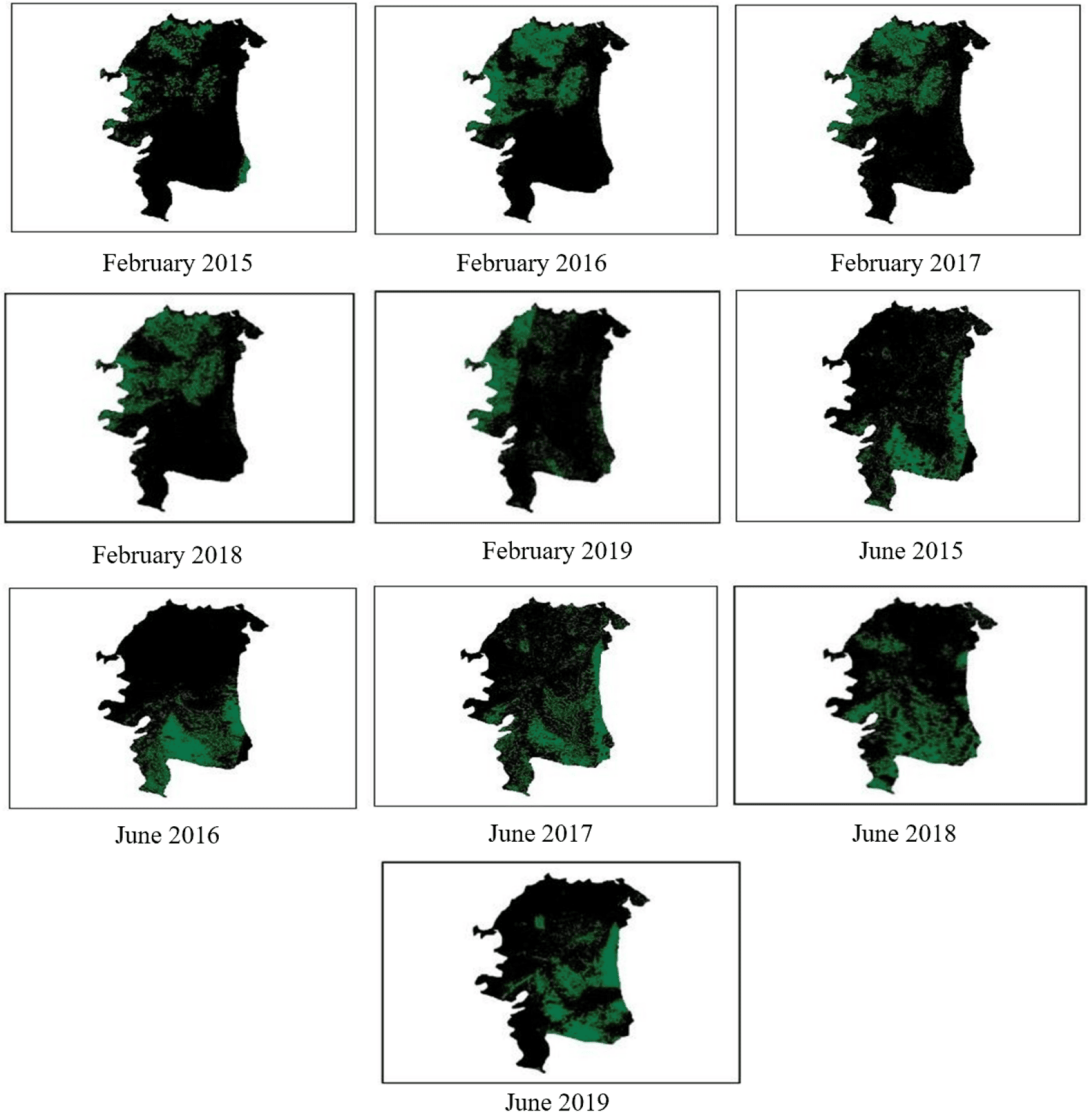
Vegetation mapping for Feb and Jun 2019 from the year 2015 to 2019.

To check for suitable land for cultivation, the process of agricultural mapping was done on Habiganj in Bangladesh to analyze the agricultural condition of the land and find out the areas where it is suitable to grow crops. Satellite images of Habiganj were processed and achieved NDVI threshold of the area based on which healthy vegetation areas during February and June were mapped as shown in Fig 11. It is a good place for cultivation due to its hilly fertile land, but its agricultural yield often fluctuates due to floods, droughts, and salinity [26]. The green areas represent the vegetation areas suitable for cultivation and K-means also show how the vegetation areas change over time. However, it is also noticed that during June- July months, due to increase in cloud coverage, inaccuracies are spotted in NDVI mapping which is one of the limitations for landsat-8.

#### E.1.2 Healthy vegetation area calculation.

**Fig 12:**
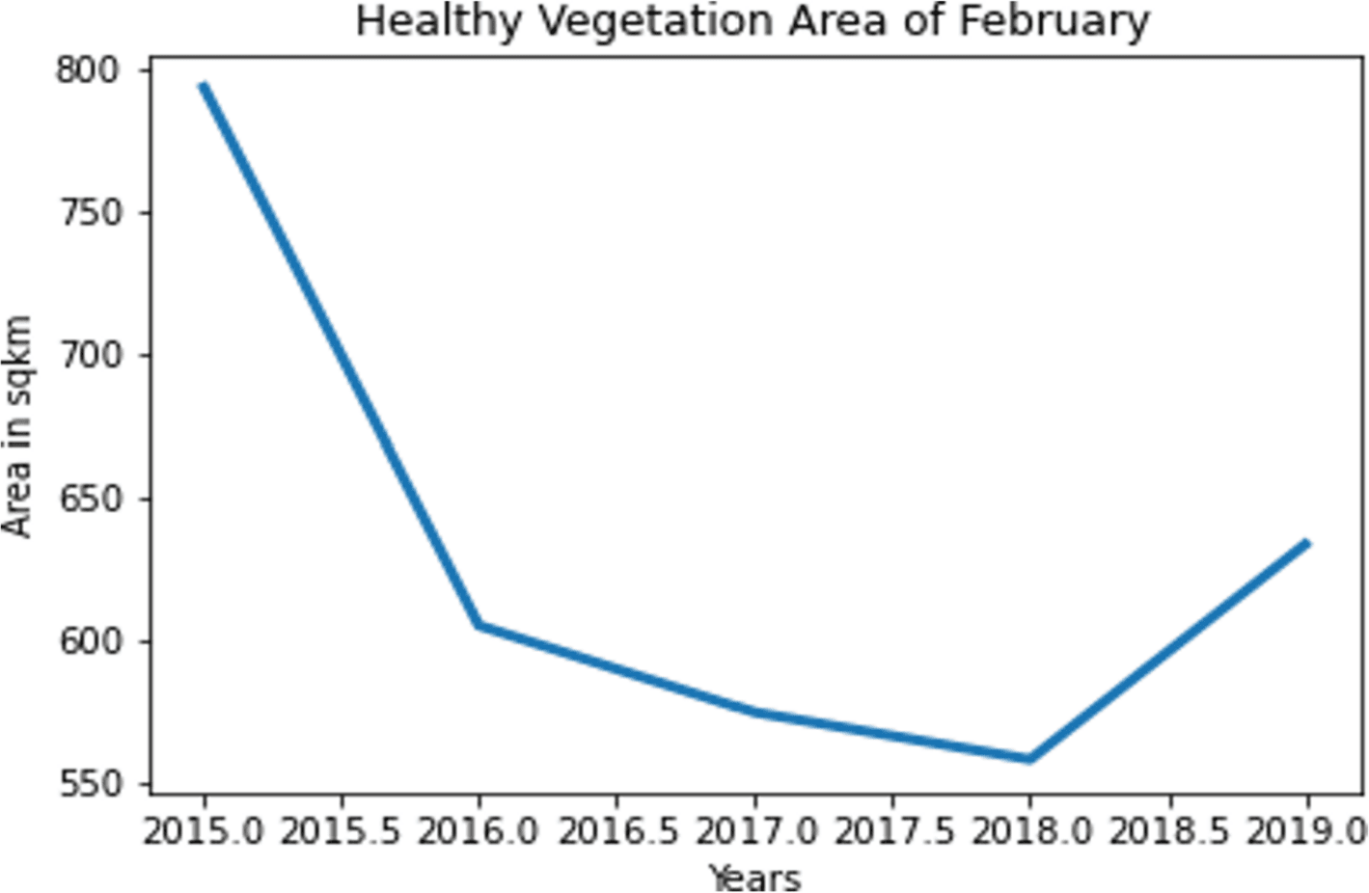
Healthy vegetation area in February from 2015 to 2019.

**Fig 13:**
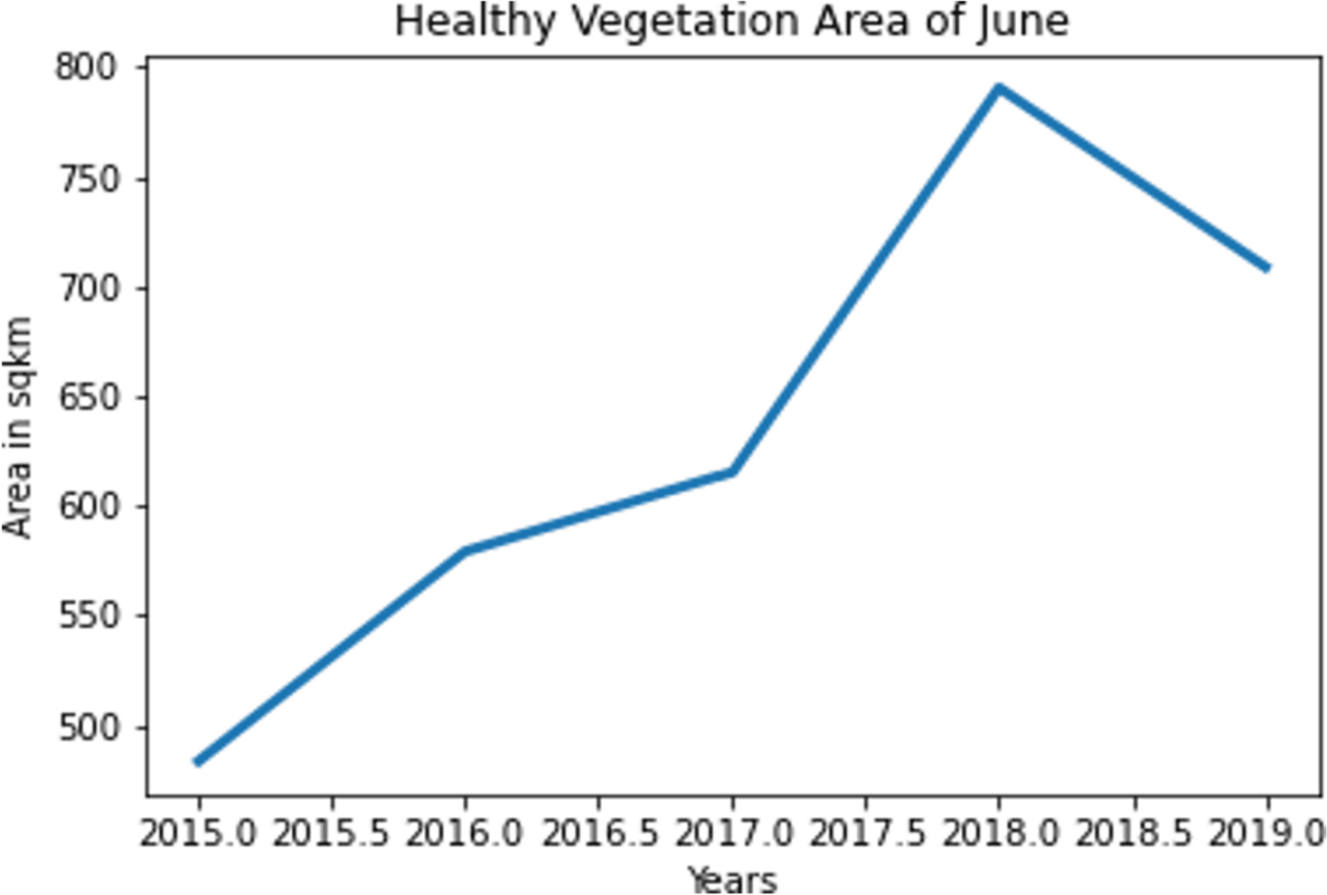
Healthy vegetation area in June from 2015 to 2019.

In order to analyze the healthy vegetation areas, the accumulated images as presented in Fig 11 is converted to binary images and only the white pixels were counted. It has been noticed that over time, there has been considerable declination in February and hence this season is generally used for minor crops which are not in high demand. As June is the monsoon period accounting to Bangladesh an exponential growth till 2018 has been noticed with in this season. That is the reason to select this season as a major harvest season. A declination in healthy vegetation is recorded in 2019 due to flood occurrence in Habiganj in that period of time. The healthy vegetation over February and June from 2015 to 2019 is presented in Fig 12 and Fig 13.

#### E.1.3 Maximum vegetation area forecasting.

**Fig 14:**
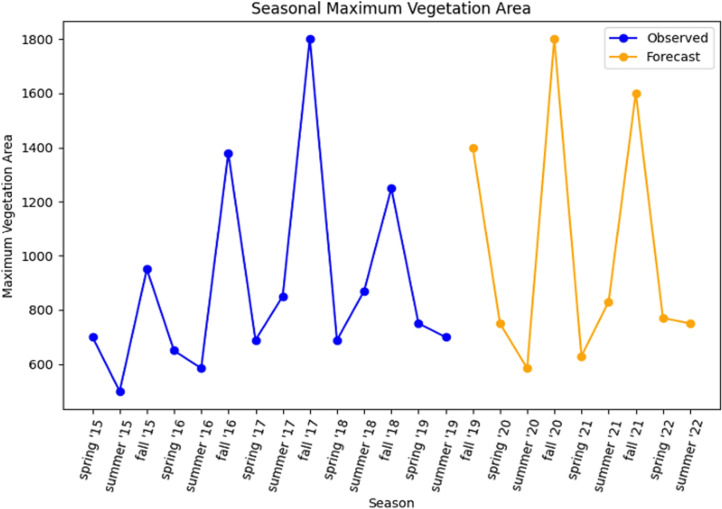
Healthy vegetation forecast for Spring, Summer, and Fall.

From the dataset, the month of February, June and November were selected for season categorization such as Spring, Summer, Fall. This categorization helps in time series model and forecasting. Using LSTM, the model is trained with 90% data as input and the rest for testing and validation. Forecasting was done for the next two years from 2019. Fig 14 shows the results of the healthy vegetation forecast for the year 2021 and 2022.

#### E.1.4 Salinity mapping.

**Fig 15:**
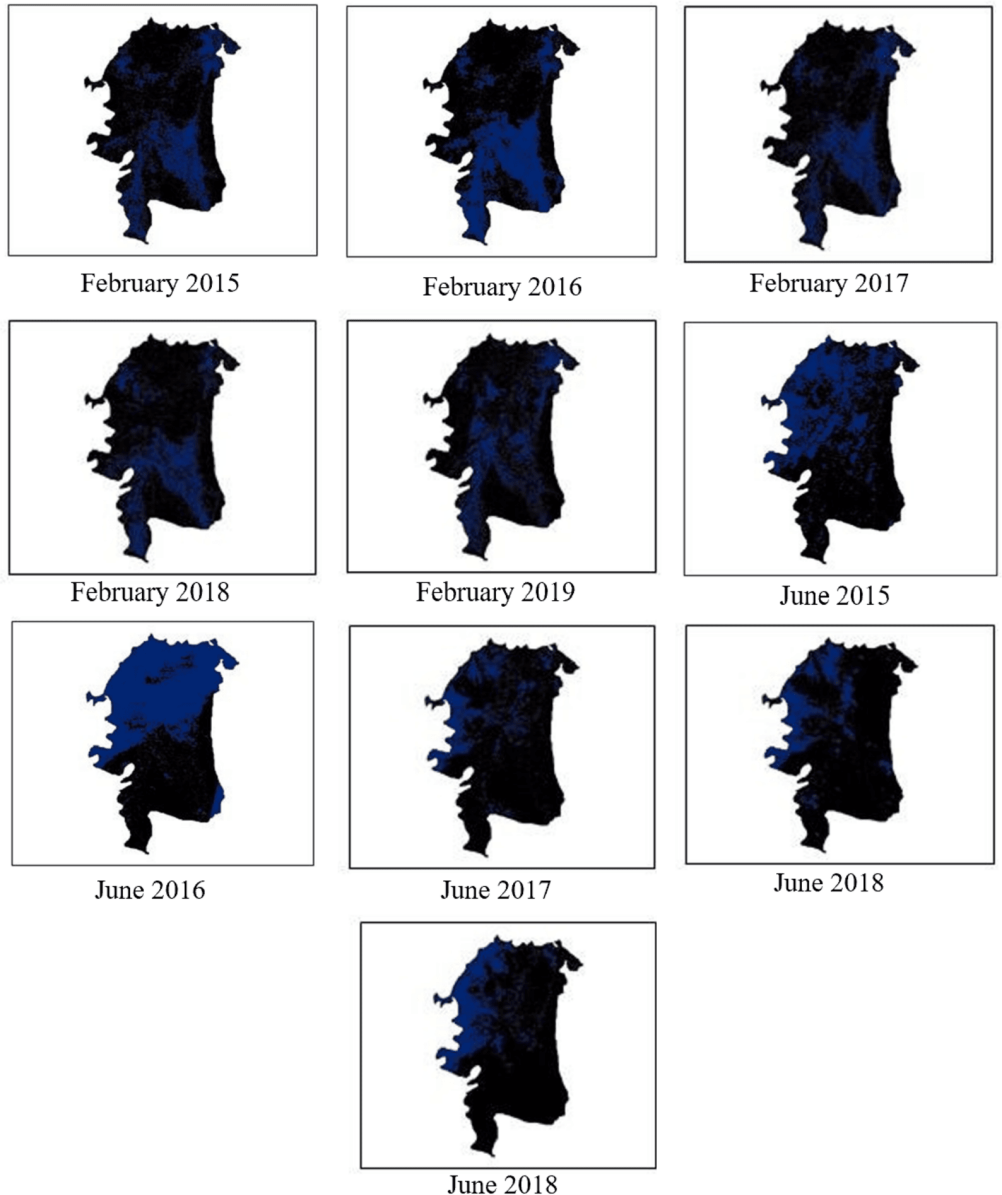
Salinity mapping for February and June from 2015 to 2019.

Using NDSI, Habiganj image is classified to high, moderate and low salinity as presented in through blue color in Fig 15. The areas with high salinity are alarming as it worsens agricultural conditions and prevents adequate crop growth. High salinity areas are highlighted using K-Mean to show which areas in Habiganj needs oversight for better yield. It can be noticed that in the month of February at 2016, there has been an increasing salinity which later diminished over time. Maximum salinity is recorded in June 2016 which signifies the salinity impact over that particular year.

#### E.1.5 Validation for crop yield prediction using different ML models.

To further determine the land arability, machine Learning algorithms such as, linear regression and random forest have been used for crop yield prediction. In Table 3 model accuracy using different loss functions are compared for crop yield prediction. The random forest model is the better-performing algorithm in this comparison, as it has higher accuracy percentages across all three-error metrics. This suggests that it captures the relationships in the data more effectively than linear regression. Additionally, a comparative analysis of different studies on the satellite image is presented in Table 4.

**Table 3:** Crop yield prediction accuracy using different ML algorithms.

Loss Function	Linear Regression	Random Forest
**MAE**	92.92%	94.86%
**MSE**	93.49%	95.87%
**RMSE**	93.49%	95.87%

**Table 4:** Comparative analysis of crop yield prediction from satellite image.

Author	Brief Title	Findings	Reference
Moussaid et al. (2023)	Tree crop yield prediction using field data and satellite imagery on Citrus Orchard	NDVI and NDWI indices, extracted from sentinel 2 and Landsat satellite images, along with other datasets were used for crop yield prediction. Several machine learning algorithms were tested for optimization. Achieved good prediction scores, 0.2489 (MAE) and 0.0843(RMSE) Shows great potential for fruit yield prediction	[41]
**A. Schwalbert et al. (2020)**	Crop yield (Soybean yield) prediction using satellite images and integrating machine learning and weather data southern Brazil	Worked on a model to forecast soybean yield using LSTM, NN, Satellite images and weather data and compared the performance of three different algorithms (multivariate OL linear regression, random forest and LSTM neural networks) for forecasting soybean yield using NDVI, EVI, land surface temperature and precipitation as independent variable LSTM neural network provides better performance relative to the other algorithms for all the forecast dates except DOY 16 where multivariate OLS linear regression delivers the best performance. Precipitation and LST were included as additional variables, which reduced the MAE, RMSE, and MSE by 16, 15, and 30%, respectively.	[42]
**Nevavuorib et al. (2019)**	Crop yield prediction with deep convolutional neural networks	Using NDVI and RGB data collected from UAVs, Convolutional Neural Networks (CNNs) are utilized to develop a model for crop yield prediction. It has been observed that while discrete error metrics yield a ranking across hyper-parameter settings, small differences in test errors can be ascribed to the stochastic nature of the optimization procedure in its whole.	[43]
**Satir and Berberoglu (2016)**	Crop yield prediction under soil salinity using satellite derived vegetation indices	Vegetation indices and Stepwise Linear Regression (SLR) from Landsat (Thematic Mapper and Enhanced Thematic Mapper) TM/ETM satellite pictures were used to estimate crop yields. When predicting the yields of wheat, corn, and cotton during the greenest part of the crop cycle, indices derived from the Landsat dataset proved to be effective. Using the Landsat dataset, this technique may be used to accurately estimate the yield two mounts before harvesting time. Field measurements taken in real time were used to confirm the predictions. The mean percent errors (MPEs) for corn, wheat, and cotton were 7.9%, 8.8%, and 6.3% respectively.	[44]
**Our system**		Extracted indices from multi-spectral images and using machine learning on the extracted dataset, conducted an agricultural analysis and crop yield prediction. Healthy vegetation areas were detected using K-means and LSTM was used to forecast healthy vegetation areas. Crop yield prediction was conducted using Linear regression and random forest algorithm where it has been observed that random forest achieves better accuracy (MAE: 94.86%; MSE: 95.87%; RMSE: 95.87%)	

### E.2 Crop and fertilizer recommendation system

#### E.2.1 ML model comparison for crop recommendations.

**Fig 16:**
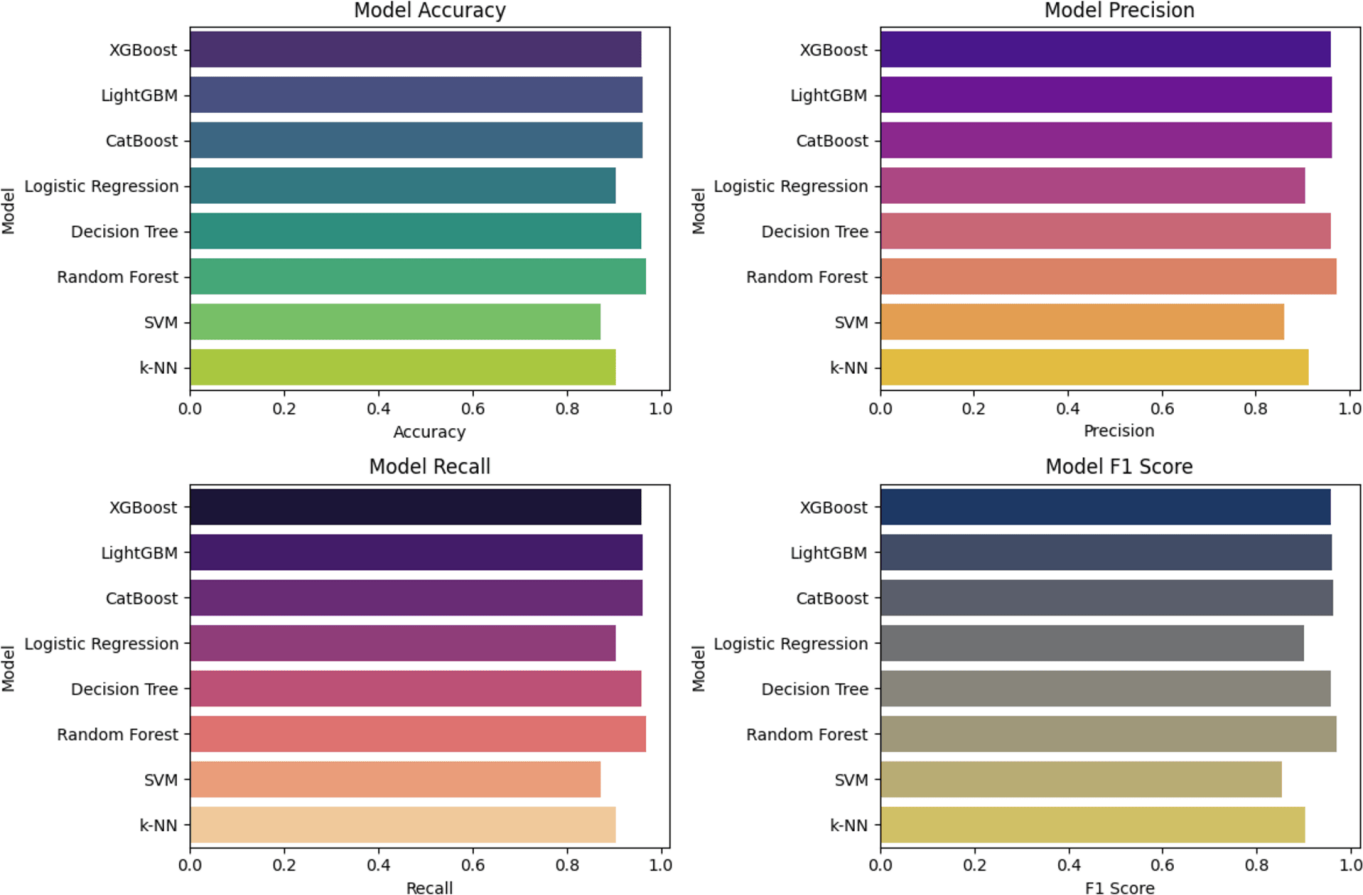
Performance comparison of ML models.

In order to predict the suitable crop based on ML algorithm the crop recommendation data set was selected [29]. Therefore, all the ML algorithms were trained on this particular data set. In Fig 16, a comparative analysis of different ML models is presented evaluating their accuracy, precision, recall and f1 score for crop recommendation. The Random Forest algorithm stands out with the highest scores across all four metrics, demonstrating its superior ability to accurately classify crops as presented in Table 5. Therefore, Random Forest is selected as the optimal model for crop recommendation, balancing accuracy and robustness, making it well-suited for practical application in agricultural decision-making.

**Table 5:** Evaluation metrics of selected ML models.

Model	Accuracy	Precision	Recall	F1 Score
XGBoost	95.75%	0.9595	0.9575	0.9572
LightGBM	96.06%	0.9618	0.9606	0.9605
CatBoost	96.21%	0.9624	0.9621	0.9618
Logistic Regression	90.30%	0.9051	0.9030	0.9017
Decision Tree	95.75%	0.9607	0.9575	0.9577
Random Forest	97.35%	0.9724	0.9735	0.9698
SVM	87.12%	0.8601	0.8712	0.8529
KNN	90.30%	0.9133	0.9030	0.9019

**Fig 17:**
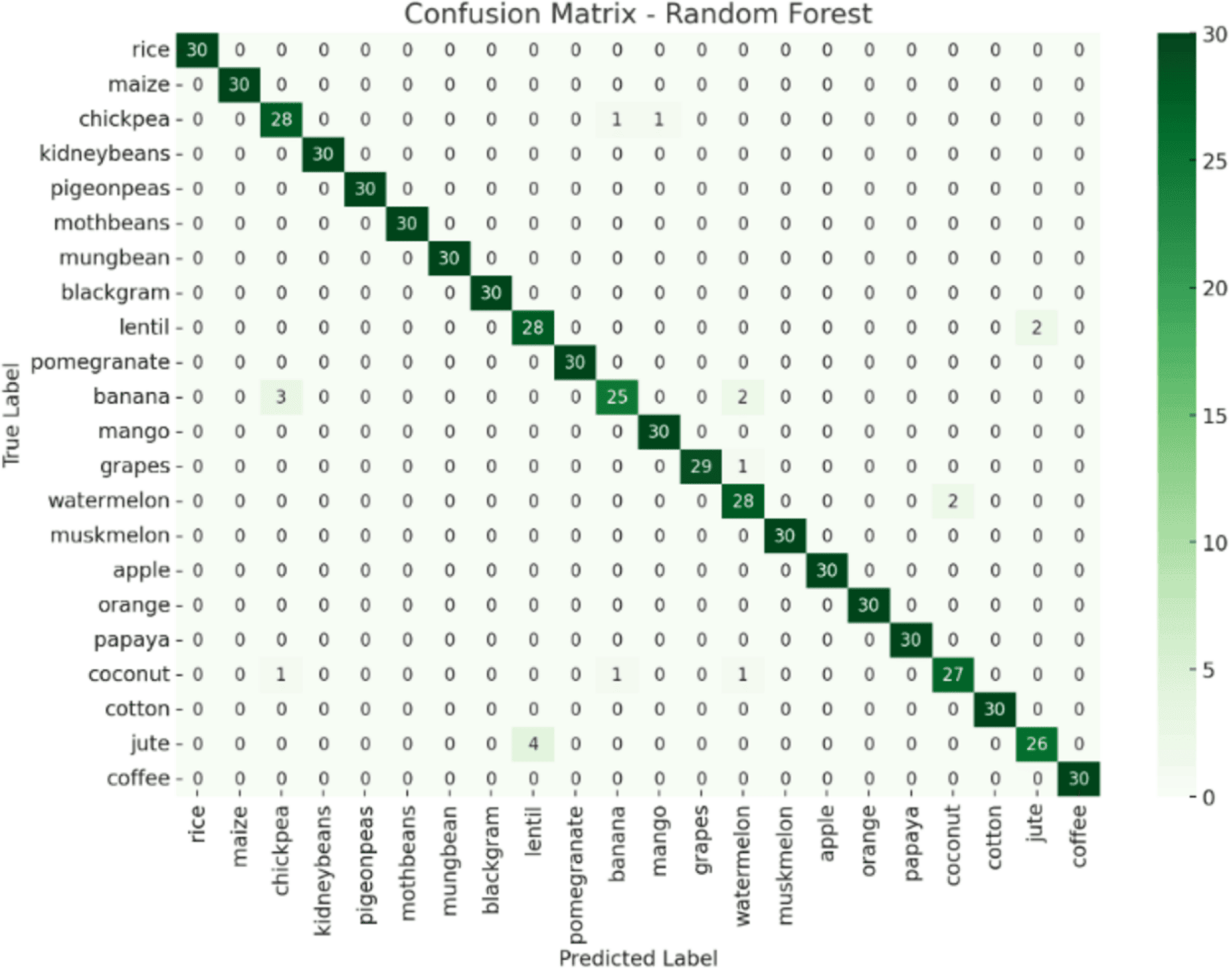
Confusion matrix of random forest model.

The confusion matrix in Fig 17 presented the reliability of a Random Forest classifier in differentiating between different crop varieties. Each cell in the matrix indicates the count of predictions generated by the model. The rows correspond to the actual classes, while the columns correspond to the predicted classes. The central diagonal indicates accurate classifications with significantly higher frequencies, while cells located off the main diagonal represent mis-classifications with lower frequencies. This visualization is important as it offers illumination into the accurate identification of crop types compared to those that are at risk of mis-classification by our selected model. According to the provided confusion matrix, it can be notices that, there remains a prediction error where for four times, jute has been predicted as lentil. Using multiple datasets might increase the system reliability.

#### E.2.2 System validation for IoT-integrated crop prediction and fertilizer recommendations.

The soil parameters are collected using the prototype. All the required parameters for suitable crop prediction and fertilizer recommendations are presented in Table 6. We can see that for all samples the temperature is $22^{o}$ C, and the humidity is 70% as that was the lab room temperature and humidity where we worked and so it is the same for all samples. Based on this laboratory environment the crop prediction was done and to further validate the prototype an outdoor test was done. It can be observed, while prototype testing, in the outdoor field the NPK parameters are similar to Tangail. Therefore, the crop prediction result is also Lentil which is also similar to Tangail.

**Table 6:** Comparative data of different soil samples.

Parameters	Laboratory soil sample test			Outdoor Test
	Barguna	Tangail	Khulna	Outdoor field
**Temperature (** ^**o**^ **C)**	22	22	22	31.8
**Humidity (%)**	70	70	70	78
**Moisture (%)**	37	52	75	62
**Soil pH**	7.8	7	7.4	7.1
**Nitrogen (mg/kg)**	190	125	224	147
**Phosphorus (mg/kg)**	65	37	189	46
**Potassium (mg/kg)**	197	132	228	154
**Crop Prediction**	Maize	Lentil	Jute	Lentil

**Fig 18:**
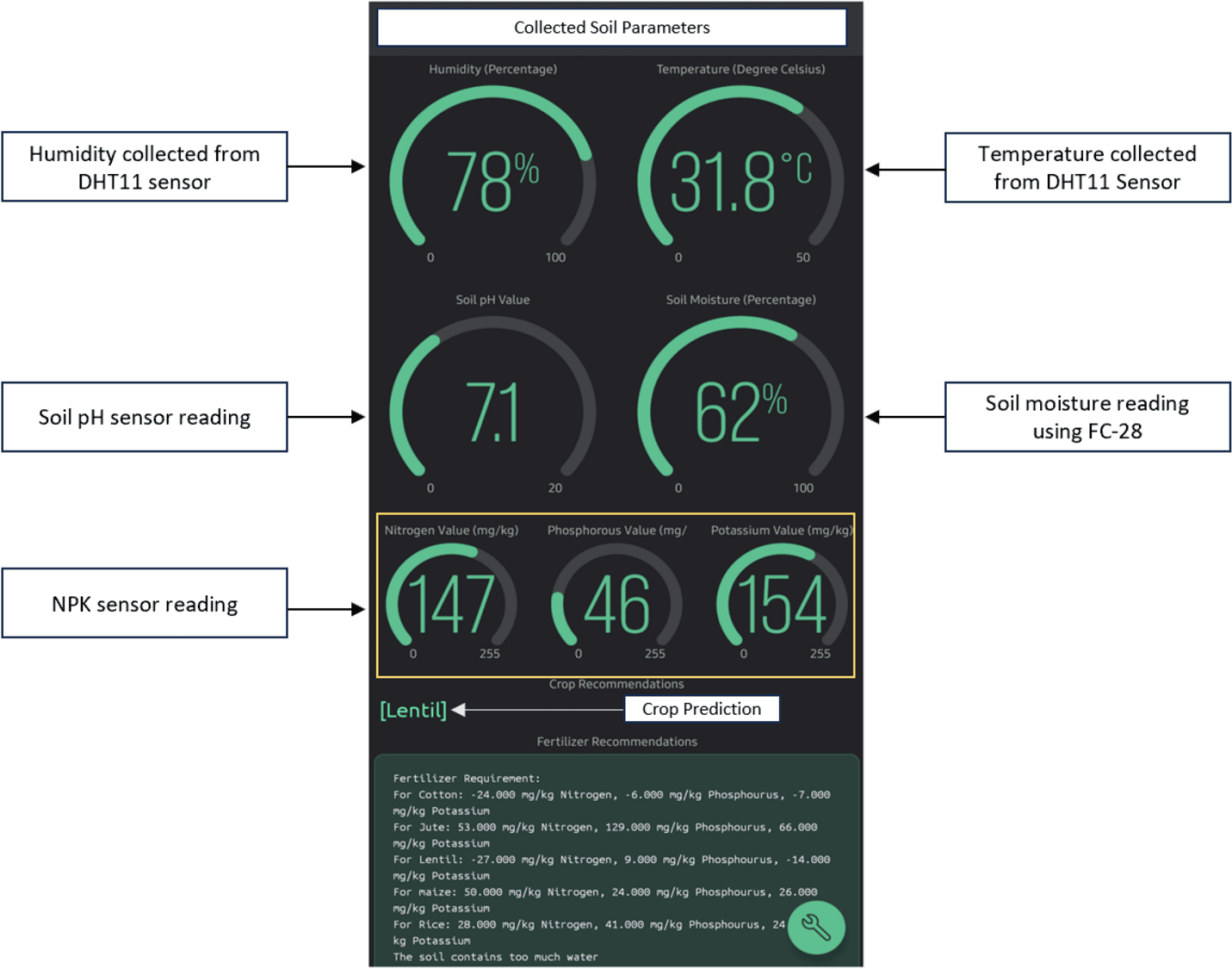
Displaying data collection and suggestions in the Blynk IoT mobile application interface.

**Fig 19:**
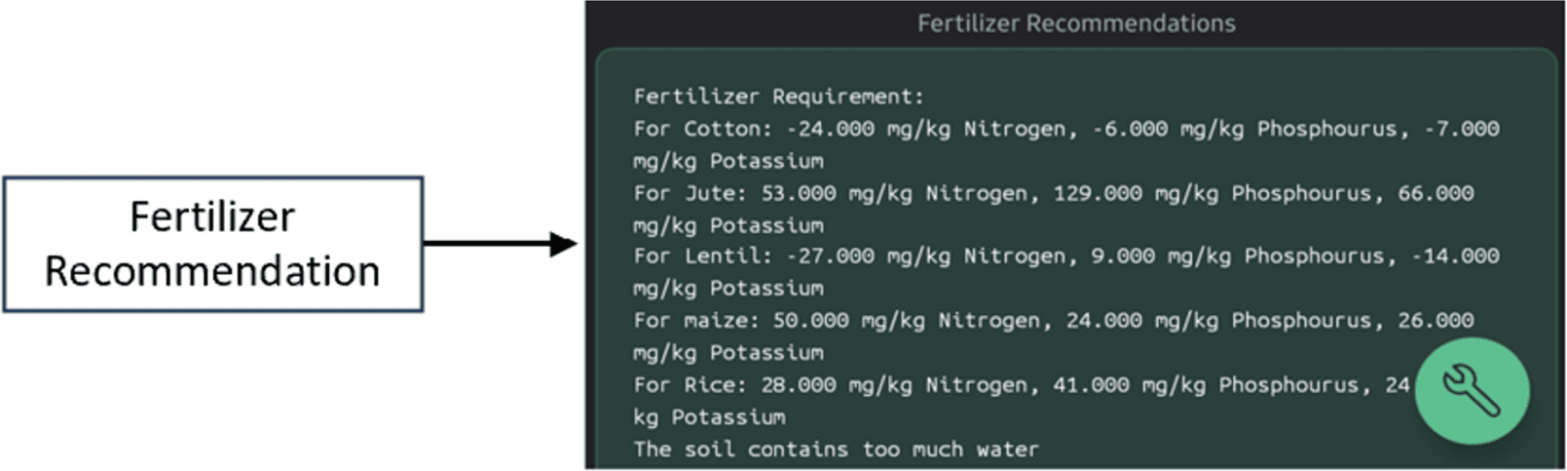
Fertilizer suggestion in Blynk IoT mobile application interface.

When these data are passed through our Random Forest model, it can predict the crop name based on the nutrient level available in the soil. The collected data from the Blynk database can be compared with the predetermined threshold values to determine fertilizer requirements. Then, we can convey the output from this cloud processing platform to the Blynk IoT application as shown in Fig 18. So, this system lets users know information such as NPK levels, humidity, pH, temperature, and soil moisture through the mobile application. Moreover, a fertilizer recommendation portion from the Blynk IoT interface is presented in Fig 19. Based on the selected crop, farmers can apply the recommended fertilizer amount after matching the value on the fertilizer recommendation chart as presented in Table 7 [28].

**Table 7:** Chart for applicable amount of fertilizers.

Fertilizer requirement by the system	Amount of fertilizer		
	Urea	TSP	MOP
Less than 0 mg/kg	N/A	N/A	N/A
10 mg/kg	7.8 kg/ha	18 kg/ha	6 kg/ha
20 mg/kg	15.6 kg/ha	36 kg/ha	12 kg/ha
30 mg/kg	23.4 kg/ha	54 kg/ha	18 kg/ha
40 mg/kg	31.2 kg/ha	72 kg/ha	24 kg/ha
50 mg/kg	39 kg/ha	90 kg/ha	30 kg/ha
60 mg/kg	46.8 kg/ha	108 kg/ha	36 kg/ha
70 mg/kg	54.6 kg/ha	126 kg/ha	42 kg/ha
80 mg/kg	62.4 kg/ha	144 kg/ha	48 kg/ha
90 mg/kg	70.2 kg/ha	162 kg/ha	54 kg/ha
100 mg/kg	78 kg/ha	180 kg/ha	60 kg/ha

Table 7 is presented considering the availability of fertilizers and the trend of fertilizer usage in Bangladesh [33]. It has been taken into consideration that Urea contains 46% nitrogen, TSP or Triple Super Phosphate contains 20% phosphorus, and MOP or Muriate of Potash contains 60% potassium creating this table. The NPK sensor provides our values in a unit of mg/kg, but we have the values for nitrogen, phosphorus, and potassium in the fertilizer recommendation guide in a unit of kg/ha. So, we have taken the help of conversion from Eq (6) [28]. A comparison table is presented in Table 8 where different research works are compared with our system.

**Table 8:** Comparison table on different studies on suitable crop prediction system.

Author	Title	ML Models	Accuracy	References
Elbasi et al.	Crop Prediction Model Using Machine Learning Algorithms	Bayes Net, Random Forest	97.05%, 97.32%	[15]
A et al.	Intelligent Crop Recommendation System using Machine Learning	Neural Network	89.88%	[45]
Kalimuthu et al.	Crop Prediction using Machine Learning	Naïve Bayes	97%	[46]
Motwani et al.	Soil Analysis and Crop Recommendation using Machine Learning	CNN	95.21%	[47]
Banerjee et al.	A Fuzzy Logic-Based Crop Recommendation System	Fuzzy logic	92.14%	[48]
Kiruthika et al.	IOT-BASED professional crop recommendation system using a weight-based long-term memory approach	IDCSO-WLSTM	92.68%	[49]
Our System		Random Forest	97.35%	

### E.3 Performance analysis of fuzzy logic and average value-based irrigation systems

For efficient irrigation we had our tests on a soil bed consisting of six soil moisture sensors and two lines of valves for both Fuzzy Logic and Average value methods. The parameters that we got from the soil bed for Average Value and for Fuzzy Logic are shown in Table 9 and Table 10 respectively. After implementing the hardware prototype, we tested a bed of 175.75 cm^2^ for four consecutive days and after implementing both the Average value and Fuzzy logic algorithm in the project, the water requirement analysis was done and compared with each other. It can be observed that the fuzzy logic-based system is 66.23% faster than the Average value control system [35]. The tabular data for operating time per days of experiment is shown in Table 11. From the measurement, with the water flow of 2.57 mL/Sec, the time required to fill a water bottle of 500 mL was 194.60 sec. Therefore, by doing the unitary method of calculation, finally the water consumption for particular amounts of area and the amount of water saved are as shown in Table 12 [35].

**Table 10:** Sensor value scenario for average value algorithm.

Sensor 1	Sensor 2	Sensor 3	Sensor 4	Sensor 5	Sensor 6	Average Value	Valve 1 Condition	Valve 2 Condition
992	985	978	975	988	991	984.8	ON	ON
990	985	978	976	987	1009	987.5	ON	ON
989	985	979	978	989	325	874.2	ON	ON
990	986	978	984	988	276	867	ON	ON
991	991	984	982	1023	296	877.8	ON	ON
991	991	984	987	140	237	721.7	ON	ON
991	991	984	981	164	261	728.5	ON	ON
990	990	983	981	174	271	731.5	ON	ON
989	988	980	293	194	292	622.7	ON	ON
988	987	979	293	194	292	622.2	ON	ON
989	987	979	293	194	292	622.8	ON	ON
990	984	980	294	195	293	622.7	ON	ON
989	201	977	295	195	292	491.5	ON	ON
989	202	976	296	196	294	492.2	ON	ON
990	203	978	297	197	295	493.3	ON	ON
1007	215	313	307	207	305	392.3	OFF	OFF
1006	215	313	308	208	305	392.5	OFF	OFF
313	212	313	309	209	306	277	OFF	OFF
313	213	313	310	209	306	277.3	OFF	OFF
310	209	309	306	206	303	273.8	OFF	OFF

**Table 11:** Run times of two algorithms.

Algorithm	Day 1 (sec)	Day 2 (sec)	Day 3 (sec)	Day 4 (sec)
**Average Value**	80.28	99.41	91.31	105.98
**Fuzzy Logic**	30.46	31.30	31.12	32.01

**Table 12:** Water consumption due to average value and fuzzy logic algorithm.

Parameters	Day 1 (mL)	Day 2 (mL)	Day 3 (mL)	Day 4 (mL)	Total (mL)
**Average Value**	206.27	255.43	243.60	272.23	968.53
**Fuzzy Logic**	78.28	60.24	79.96	70.66	289.14
**Saved Water**	127.9	195.19	154.64	201.65	679.39

**Table 9:** Sensor value scenario for fuzzy logic algorithm.

Sensor 1	Sensor 2	Sensor 3	Sensor 4	Sensor 5	Sensor 6	Fuzzified Value 1	Fuzzified Value 2	Valve 1 Condition	Valve 2 Condition
992	984	977	974	988	991	32	32	ON	ON
991	984	997	974	988	991	32	32	ON	ON
992	985	978	975	988	991	32	32	ON	ON
990	985	978	976	987	1009	32	32	ON	ON
989	985	979	978	989	325	32	32	ON	ON
990	986	978	984	988	276	32	32	ON	ON
991	991	984	982	1023	296	32	32	ON	ON
991	991	984	987	140	237	32	32	ON	ON
1009	205	1001	298	198	296	32	0	ON	OFF
1002	203	297	296	196	293	32	0	ON	OFF
1004	209	304	301	201	298	32	0	ON	OFF
1005	210	307	303	203	300	32	0	ON	OFF
1006	211	309	304	204	301	32	0	ON	OFF
1005	212	309	305	204	302	32	0	ON	OFF
1006	212	310	305	205	302	32	0	ON	OFF
211	120	217	216	116	212	0	0	OFF	OFF
213	121	219	218	118	213	0	0	OFF	OFF
214	122	220	218	118	214	0	0	OFF	OFF
215	123	221	220	119	215	0	0	OFF	OFF
216	124	222	221	120	216	0	0	OFF	OFF

**Fig 20:**
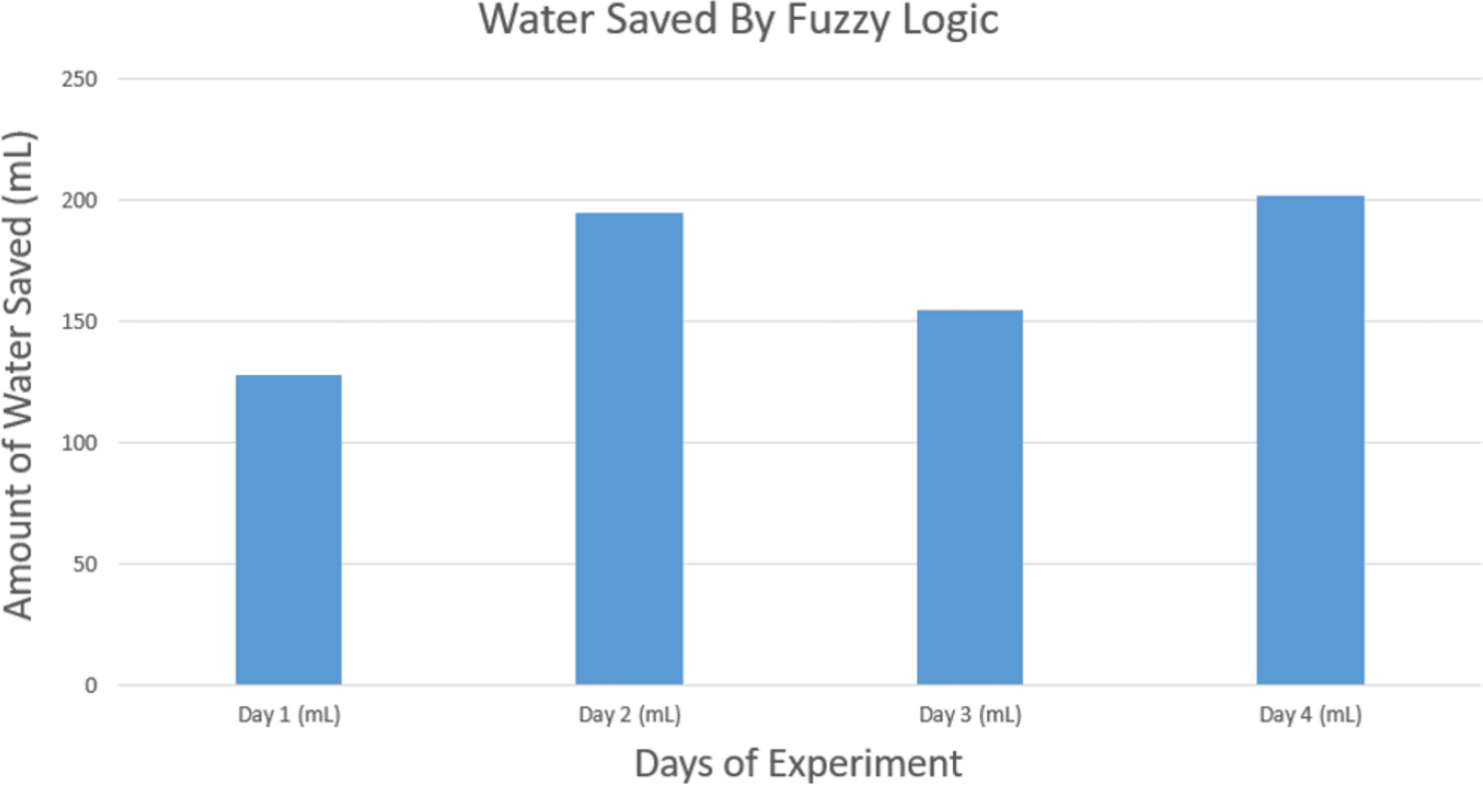
Graphical representation of water saved by Fuzzy Logic.

From the calculations, the around 679.39 mL of water is saved using the fuzzy logic in comparison to average value in four days of the experiment. Fig 20 presents the amount of water saved in day-to-day use. The fuzzy logic algorithm for the irrigation system saves almost 70% of water compared to the Average Value method while ensuring accurate amounts of water needed for crops. The irrigation presented here is a prototype that was implemented in a limited space of 175.75 cm^2^ soil bed. Previously, there has been work on irrigation systems by Krishnan et al. using a fuzzy logic system which reduces around 45% water usage [22]. Benyezza et al. was able to save 19% water usage using fuzzy logic compared to the conventional irrigation systems [50]. Meanwhile, our implemented system is able to save 70% of water.

### F.1 Economic feasibility of the proposed system

The proposed system is segmented in to three parts which are remote sensing through satellite images, system implementation for crop and fertilizer recommendation and hardware set up for efficient and cost-effective irrigation system. Here, hardware implementation was required for crop and fertilizer recommendation system and irrigation system. A detailed cost analysis on the hardware system is provided in Table 13 and Table 15.

**Table 13.** Cost analysis for prototype implementation for crop and fertilizer recommendation system.

Components Name	Price/Unit	Quantity	Total Price
ESP-WROOM- 32	$15.99	01	$15.99
YL-69 Soil Humidity & Soil Moisture Sensor	$1.50	01	$1.50
Soil NPK Sensor	$ 89.41	01	$ 89.41
Soil pH Sensor	$64.91	01	$64.91
DHT 11	$5.99	01	$5.99
LiPo Battery (2200mAh)	$28.50	01	$ 28.50
Buck Boost Converter	$11.99	02	$ 23.98
Others			$5.00
Total			$235.28

#### F.1.1 Cost analysis for crop and fertilizer recommendation system.

In developing countries, there is always an option to test the soil parameters for analyzing the soil nutrients and categorize that for the suitable crop cultivation. But with the delayed functionality of this process, the system becomes impractical for most of the farmers. Our proposed crop and fertilizer recommendation system works on real time and the results can be seen on the phone screen for better visualization and complete suggestion. The cost of the major components of the prototype is presented in Table 13. Minor components like MOSFETs, switches, resistors are not presented here.

#### F.1.2 Cost analysis for fuzzy logic-based solar-powered irrigation system.

The developed system is a hybrid system, with solar power serving as the primary energy source to operate the entire setup. To execute this, solar pumps are incorporated, which offer significant long-term cost-saving benefits. Unlike traditional diesel engine pumps commonly used by farmers, solar pumps are more economical over time. A detailed comparison between these two types of pumps is presented in Table 14.

**Table 14:** Cost comparison between diesel pump and solar pump.

Pump Parameters	Diesel Pump	Solar Pump (Centrifugal)
**Power**	1HP	1HP
**Discharge**	150,000 L/day by 760 mm discharge pipe	150,000 L/day by 760 mm discharge pipe
**Diesel consumption**	1,440 L/year	N/A
**Operation time** **(4 h/day, 3 crops per year)**	4,380 h	4,380 h
**Life span**	10 years with engine overhauling after 5 years	25 years with possibly no overhauling
**Installation cost**	US$ 512	US$ 769

According to our study, the price of diesel fuel is USD 0.946 per liter. Based on calculations, the annual diesel consumption for a diesel pump amounts to USD 1,362.24. In contrast, the hybrid solar-powered centrifugal pump proves to be far more practical and cost-effective than the diesel engine pump. Having a lifespan of 25 years, this solar-based pump is predicted to save approximately USD 34,056 over its lifetime. A detailed cost analysis on the efficient irrigation control system is provided in Table 15.

**Table 15:** Cost analysis of irrigation controller unit.

Components Name	Price/Unit	Quantity	Total Price
Arduino MEGA 2560	$10.96	02	$21.92
YL-69 Soil Humidity & Soil Moisture Sensor	$1.50	50	$75
LDR Sensor	$0.75	02	$1.5
NRF24L01 Wireless Transceiver Module	$2.31	02	$4.62
Ultrasonic sensor HC- SR04	$1.15	01	$1.15
LCD Display 16*2 with Female Header	$2.02	01	$2.02
2 Channel 5V Relay Module	$1.44	05	$7.2
12 v Solenoid Valve	$5.19	10	$51.9
Others			$4.69
Total			$170

This prototype is designed for a large-sized bed, providing a clear understanding of the cost for implementation. The cost of the controller is minimal when compared to the long-term savings in water. Preserving natural resources, such as groundwater, for future sustainability is a significant benefit of this system.

## Conclusion and future work

Agriculture plays a pivotal role in sustaining human life and economic sustainability. This research has incorporated image processing, IoT, machine learning and fuzzy logic control systems to modernize agricultural practices. With the help of multi-spectral satellite images and agricultural mapping, the system can determine the suitability of a land for farming. The IoT-based framework for real-time soil monitoring provides crop recommendations and optimal fertilizer requirements. Through the implementation of fuzzy logic-controlled solar irrigation system, the water usage is optimized. This results in a significant reduction in water and electricity consumption. The k-mean and LSTM algorithms were applied to satellite image data obtained from 2015 to 2019 to forecast the healthy vegetation area from 2020 to 2022. Therefore, machine learning algorithms were integrated for crop yield prediction. The effectiveness of remote sensing combined with advanced computational techniques to enhance agricultural productivity is presented in this article. Multiple machine-learning methods are utilized for the crop recommendation system. The random forest algorithm achieved the highest accuracy rate of 97.35%, indicating that it is a suitable option for implementation. Meanwhile, due to the fuzzy logic control system, almost 70% of water is saved. Solar panels are becoming more affordable, making them a more sustainable and economically viable investment choice. The utilization of solar-powered irrigation systems can greatly reduce a country’s electricity demand, considering that irrigation requires a considerable amount of energy. This change not only facilitates the adoption of sustainable agriculture practices but also fosters energy preservation and economic stability. A routine check can be done for vegetation, crop, and fertilizer recommendations during cultivation to understand and predict the change of vegetation and suitable crop in the upcoming months and seasons and farm accordingly.

By means of increasing the dataset and integrating more varied geographical areas and different climate conditions, the system’s robustness can be enhanced. Including real-time weather forecasting data will help with better irrigation plans. Due to budget constraints, the fuzzy logic-based irrigation system was not implemented in field. So, for future direction it is imperative to apply the irrigation system and test in real field so that it can be compared with other research works. Further investigation on the scalability and cost impact of the proposed technology in diverse agricultural scenarios is essential to understanding the economic viability and potential of the system.
